# Defiant: (DMRs: easy, fast, identification and ANnoTation) identifies differentially Methylated regions from iron-deficient rat hippocampus

**DOI:** 10.1186/s12859-018-2037-1

**Published:** 2018-02-05

**Authors:** David E. Condon, Phu V. Tran, Yu-Chin Lien, Jonathan Schug, Michael K. Georgieff, Rebecca A. Simmons, Kyoung-Jae Won

**Affiliations:** 1 Department of Genetics, The Institute for Diabetes, Obesity, and Metabolism, Perelman School of Medicine, University of Pennsylvania, Philadelphia, PA 19104 USA; 2 Department of Pediatrics, University of Minnesota, 2450 Riverside Avenue, Minneapolis, MN 55454 USA; 3 Center for Research on Reproduction and Women’s Health, University of Pennsylvania, 421 Curie Blvd, Philadelphia, PA 19104 USA; 4 Biotech Research and Innovation Centre (BRIC), University of Copenhagen, 2200 Copenhagen, Denmark

**Keywords:** Epigenetics, DNA Methylation, WGBS, Differentially Methylated regions (DMR), RRBS, Bisulfite sequencing

## Abstract

**Background:**

Identification of differentially methylated regions (DMRs) is the initial step towards the study of DNA methylation-mediated gene regulation. Previous approaches to call DMRs suffer from false prediction, use extreme resources, and/or require library installation and input conversion.

**Results:**

We developed a new approach called Defiant to identify DMRs. Employing Weighted Welch Expansion (WWE), Defiant showed superior performance to other predictors in the series of benchmarking tests on artificial and real data. Defiant was subsequently used to investigate DNA methylation changes in iron-deficient rat hippocampus. Defiant identified DMRs close to genes associated with neuronal development and plasticity, which were not identified by its competitor. Importantly, Defiant runs between 5 to 479 times faster than currently available software packages. Also, Defiant accepts 10 different input formats widely used for DNA methylation data.

**Conclusions:**

Defiant effectively identifies DMRs for whole-genome bisulfite sequencing (WGBS), reduced-representation bisulfite sequencing (RRBS), Tet-assisted bisulfite sequencing (TAB-seq), and HpaII tiny fragment enrichment by ligation-mediated PCR-tag (HELP) assays.

**Electronic supplementary material:**

The online version of this article (10.1186/s12859-018-2037-1) contains supplementary material, which is available to authorized users.

## Background

DNA methylation plays a critical role in gene regulation [1]. In human somatic cells, 70–80% of all CpG dinucleotides in the genome are methylated [2]. DNA methylation represents one type of epigenetic modification which has been shown to control transcription in mammals [3], interacting sometimes with DNA binding proteins [4]. DNA methylation regulates many diverse biological functions, such as embryonic stem cell differentiation [5], aging [6], gene imprinting [7, 8], and X-chromosome inactivation [9]. DNA methylation is conserved and somatically heritable mark that is generally associated with transcriptional repression [10]. Aberrant methylation has been found in multiple diseases such as cancer [11], imprinting defects [12] and mental disorders such as schizophrenia [13, 14]. Environmental exposures such as uteroplacental insufficiency [15, 16] or cigarette smoking [17, 18] have also been observed to alter DNA methylation.

Recent developments in sequencing technology enabled genome-wide characterization of DNA methylation. Whole-genome bisulfite sequencing (WGBS) and reduced representation bisulfite sequencing (RRBS) have been widely used to measure DNA methylation at a single CpG resolution [19]. DMRs are the contiguous genomic regions whose DNA methylation status differs between two groups of samples. DMRs have been used to characterize cell-type or condition specific DNA methylation [20–22].

Computational approaches have been developed based on statistical frameworks to identify DMRs. BSmooth [23] identifies DMRs using a local-likelihood approach to estimate a sample-specific methylation profile. For a statistical test between samples, BSmooth uses a Welch’s t-test [24]. MethylKit identifies DMRs based on logistical regression if multiple replicates are available, or a Fisher’s exact test [25] if only one sample is available. MethylSig [26] uses a beta-binomial approach to identify DMRs based on read coverage and biological variability, which showed high sensitivity in comparison with MethylKit, BSmooth, a standard t-test and the Wilcoxon rank test. Metilene [27] uses a binary segmentation algorithm combined with a two-dimensional Kolmogorov-Smirnov test. Genomic regions are pre-segmented, and gradually reduced in size until the region contains less than a defined minimum number of CpGs or statistical significance is not improved. RADMeth [28] employs a beta-binomial regression and a Stouffer-Liptak test. And RnBeads [29] calculates *p*-values for each CpG in the data set using hierarchical linear models and M-values [30]. BiSeq uses a smooth-based approach while considering coverage to call DMRs [31].

With the exception of Metilene, many well-known DMR callers require knowledge of the R programming language [32] or even a specific version of R (MethylKit & BiSeq). In addition, each DMR caller requires specific input formats to run it properly. Furthermore, many of them use extreme computing resources for genome-scale analysis. Table 1 summarizes the characteristics and algorithms of the widely used DMR callers we investigated.

**Table 1:** Comparison of DMR calling software

Program	DMR Identification	Execution
Defiant	Weighted Welch Expansion	Binary
BSmooth [23]	Local-likelihood smoothing with binomial test	R
MethylKit [25]	Fisher’s exact test [37] or logistic regression with tiling	R
MethylSig [26]	beta-binomial [64]	R
Metilene [27]	*p*-value by beta binomial	Binary
MOABS [65]	beta-binomial	Binary
RADMeth [28]	beta-binomial regression	Binary

The numerous disadvantages of these programs/statistical methods prompted the development of a new DMR-identification program. Defiant is a standalone program following GNU99 standard, which reduces the issues of portability. Defiant automatically detects ten different input formats widely used for DNA methylation. It does not require installation of any libraries and runs with a single command line. We evaluate Defiant’s performance with comprehensive benchmarking tests using both artificial and real WGBS data. We applied Defiant to analyzing the DNA methylation changes induced by iron deficiency during the critical neuro-developmental period (fetus and newborn) in the rat hippocampus.

## Implementation

### Animals and Hippocampal dissection

G2 pregnant CD1 Sprague-Dawley rats were purchased from Charles Rivers Laboratories. The experimental conditions for induction of fetal-neonatal iron deficiency were following the previously described protocol [33]. All procedures were approved by the Institutional Animal Care and Use Committee of the University of Minnesota. Hippocampal dissection and storage from PND15 rats was performed as previously described [33]. Genomic DNA from PND15 rat hippocampi was isolated using Allprep DNA/RNA mini kit (Qiagen).

### WGBS & data processing

WGBS was performed as a published protocol [34]. Briefly, 1 μg of genomic DNA was fragmented into 300 bp size using M220 Covaris Ultrasonicator. Sequencing libraries were generated using NEBNext genomic sequencing kit (New England Biolabs) and ligated with Illumina methylated paired end adaptors. Libraries were bisulfite-converted using Imprint DNA modification kit (Sigma), and the size of 300–600 bp was selected using Pippin Prep DNA size selection system (Sage Science). Libraries were then amplified using PfuTurbo Cx Hotstart DNA polymerase (Agilent Technologies). Samples were sequenced to 100 bps in either paired-end or single-read formation on an Illumina HiSeq 2000 with RTA version 1.13.48 and HiSeq control software version HiSeqCS:1.5.15.1. Adpaters were trimmed from the reads using a custom C language program. Trimmed reads were aligned against the rat genome (rn4) using bs seeker (v1) [35]. The methylation status was then tallied from the bs seeker output. When reads overlapped at a base, the methylation status from read 1 was used. Methylation data at the C and G in a CpG pair was merged to produce the estimate at that locus. The WGBS data have been deposited in the GEO repository (GSE98064).

### Identification of DMRs using WWE

Defiant defines DMRs based on seven criteria. These can also be specified by the end-user.

- All nucleotides in all samples are present and meet minimum coverage (default 10). The user can specify that some nucleotides can be missing from certain replicates, but we recommend against it as it can introduce false positives.
- Absolute value in the difference of the sum of the methylation percentages is above a given cutoff %. The default is 10%, but this will vary in every individual experiment based on the chemistry [36]. The mean methylation percentage, $\overline{m}$, is weighted based on coverage, i.e.
  1 $$ \overline{m}=\frac{\sum_{r=1}^R{C}_r\frac{{}_m{C}_r}{C_r}}{\sum_{r=1}^R{C}_r}=\frac{{\sum_{r=1}^R}_m{C}_r}{\sum_{r=1}^R{C}_r} $$
  where *C*_*r*_ is the coverage for replicate *r*, _*m*_*C*_*r*_ is the number of 5mC for replicate *r*, and *R* is the number of replicates.
- A 2-tailed *p*-value, default 0.05

If there is only 1 replicate in either sample, a *p*-value between groups A and B is calculated by Fisher’s exact test [37]

2 $$ p=\frac{\left({}_m{C}_A +_m{C}_B\right)!\left({C}_A+{C}_B\right)!\left({}_m{C}_A+{C}_A\right)!\left({}_m{C}_B+{C}_B\right)!}{{}_m{C}_A!{}_m{C}_B!{C}_A!{C}_B!\left(\Big({}_m{C}_A +_m{C}_B+{C}_A+{C}_B\right)!} $$

where _*m*_*C =* number of 5-methyl Cystosine and *C* = number of Cytosine.

If there are multiple replicates in both groups, the *p*-value is based on Welch’s t-test [24] in sum of methylation percentages is below a given cutoff.

If both samples have multiple replicates, the *t*-test between groups A and B is calculated thus:

3 $$ t=\frac{{\overline{m}}_B-{\overline{m}}_A}{\sqrt{\frac{s_A^2}{N_B}+\frac{s_B^2}{N_B}}}, $$

where the unbiased sample variance *s* for any group *A* is also weighted based on coverage:

4 $$ {s}_A=\frac{\sum \limits_{r=1}^R{C}_r{\left({\overline{m}}_A-{m}_r\right)}^2}{\left(\sum \limits_{r=1}^R{C}_r\right)-1} $$

The Benjamini-Hochberg [38] approach is applied to the identified DMRs to adjust *p*-value for multiple testing.

- a minimum number of CpN constituting differentially methylated region (default CpN = 5),
- a minimum range of the differentially methylated nucleotides (default 0)
- a maximum range between CpN (default 20,000 nucleotides).
- a maximum similar, i.e. non-differentially methylated, CpG count (default 5). If a DMR is currently expanding, the DMR expands until one criterion of differential methylation stops. By allowing a similar CpG count, DMRs can contain similarly methylated CpG inside the DMR. Once the similar nucleotide is broken, the DMR shrinks to the point where differential methylation stopped.

All criteria are easily set by command-line options. Defiant is designed to test multiple parameters in parallel, to make the final decision on each parameter up to the end user. Defiant has a companion program in Perl, plot_results.pl, which plots the number of DMRs as a function of different parameters using GNUPlot if a user has chosen to test multiple parameters. The effects of the variation of each parameter on the number of DMRs found in the rat hippocampus data can be seen in (Additional file 1: Figures S2-S16).

The number of DMR is sensitive to CpN, differentially methylated CpN (d), and *p*-value, but less sensitive to minimum percent change, minimum coverage. Minimum CpN = 5 is a point where the number of DMR is large enough and less sensitive to minimum coverage (Additional file 1: Figure S7). Minimum coverage determines the number of DMRs. The default (10) is the point where the number of DMR is robust to the “Differentially methylated CpN” (Additional file 1: Figure S8). We set a large number for “Maximum range for CpN” (default = 20,000). The difference in the methylation percentage (default 10%) can further be used to identify subtle but significant changes. A minimum range between CpN is not used for the rat data (default 0) but provide a user with flexibility in defining DMRs.

### Comparison with other methods

**Fig. 1:**
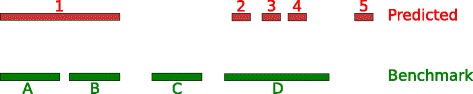
Illustration of how different DMR results are compared. Artificial (or “Benchmark”) DMRs are depicted in green, while predicted DMRs are shown in red

DMR Overlay is the percent of nucleotides predicted by the program inside of the artificial DMR. DMR overlay is measured in two directions, once with respect to the benchmark DMRs and once with respect to the predicted DMRs (Fig. 5). For example, in Fig. 1, the overlay of predicted DMR 1 with respect to Benchmark DMR “A” is calculated as

$$ \mathrm{DMR}\ \mathrm{overlay}=\frac{\mathrm{length}\left(\mathrm{A}\right)+\mathrm{length}\left(\mathrm{B}\right)}{\mathrm{length}\ \left(\mathrm{DMR}\ 1\right)}=\frac{160+139}{319}=93.7\% $$

while for the reverse, DMRs A and B would each have their own DMR overlay equal to 100%. This is similar, but more informative than a Jaccard index, which is symmetric while DMR overlay depends on direction of comparison.

- Defiant is run with default minimum coverage of 10, *p* = 0.05, minimum percent change of 10%, and five CpN (CpG) in each DMR.
- MethylKit [25] scans for DMRs using a tiling window of 1000 nucleotides and a step size of 1000 nucleotides. Consecutive windows that score q < 0.05 are considered as a single DMR. Tiles can report q = 0 with p ≈ 1, so tiles with q = 0 are treated as non-DMRs. MethylSig [26] also uses a tiling approach, with a window and step size of 25 nucleotides. As for MethylKit, consecutive windows with q < 0.05 are considered as a single DMR.
- Metilene [27] is a command-line program, and was run according to defaults. Metilene was run with a minimum CpG count of 5 with the rat data to make results comparable to Defiant.
- RADMeth [28] is run as a series of three commands to the Linux shell. RADMeth showed itself superior to ComMet [39] and DSS [40] so we do not compare these methods here.
- RnBeads [29] differentially methylated regions are defined by 500 nucleotide tiles. Similarly with MethylKit and MethylSig, consecutive tiles with q < 0.05 are considered as a single DMR.

## Results

### DMR identification by weighted Welch expansion

Defiant calculates a *p*-value using a Welch’s t-test for the weighted means and variance (Method). A weight is to give more credence to the replicate with a high coverage. If there is only one replicate in either set, Welch’s t-test cannot be used, and thus Defiant uses a Fisher’s exact test. For accurate detection of the boundary of a DMR, Defiant detects the start point based on the following factors: differences in methylation levels, coverage, *p*-value, and a minimum number of CpGs. If all of these criteria are satisfied, the next nucleotide is checked for constituting differential methylation, i.e. the DMR expands. It terminates when there is a number of consecutive CpGs that do not pass the criteria. We call this algorithm weighted Welch expansion (WWE).

**Fig. 2:**
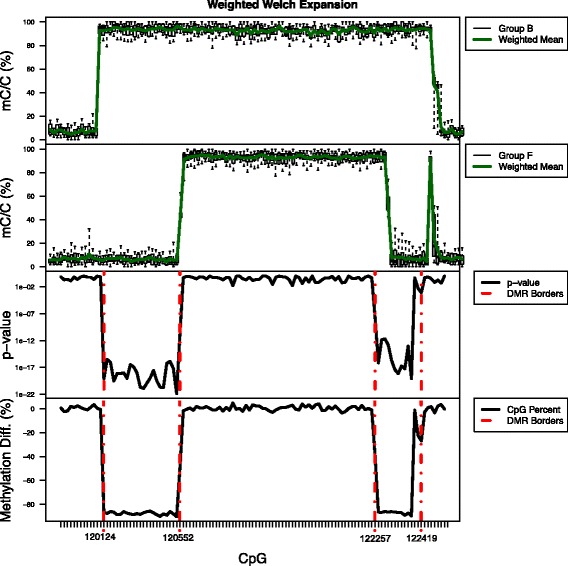
Defiant uses WWE for DMR identification. We used artificial data designed for Metilene [27]. Each group is composed of ten replicate DNA methylation samples. The top two panels show the level of DNA methylation for each CpG (box-and-whisker plots). The mean is weighted based on coverage. The third panel from the top shows the weighted Welch *p*-value between the sets for individual CpG. The bottom panel shows differences between the weighted mean. Defiant calls a DMR when it finds consecutive CpGs with 1) differences in methylation levels, 2) minimum coverage, 3) *p*-value. When Defiant finds consecutive CpGs that do not match the above criteria, the expansion of a DMR stops. The third and fourth panels show the DMR start and end points in red

Figure 2 demonstrates how Defiant identifies DMRs using WWE. Defiant calculates the weighted mean based on the coverage. The weighted mean for the two datasets (“B” and “F”) are shown on the top two panels. The *p*-values were calculated using a Welch’s t-test (on the third panel). The DMR on the left side starts at 120,124 when the difference in methylation levels is − 88% and *p* = 7.7 × 10^− 20^. The DMR expands as long as it sees CpGs that pass the criteria. At 120,052, Defiant stops its expansion when it observes at least 5 consecutive CpGs that fail the criteria. Defiant does not require setting the size of a window or “tiles” as is done in MethylKit [25], so the data determines the size of the differentially methylated regions. This approach gives much greater flexibility and power in analyzing the data, as small differentially methylated regions can be easily missed when using a tiling approach.

### Datasets

We used two datasets to evaluate Defiant’s performance.

#### Artificial benchmarking datasets used for Metilene [27]

Metilene [27] simulated RRBS and WGBS datasets using beta binomial distribution. The dataset are composed of two different backgrounds, each with four subsets. These are named 1.1 (strongest methylation differences) through 1.4 (weakest differences) for the first background, and 2.1 through 2.4 for the second (Additional file 1: Figure S1). In total, there are 16 subsets (eight for RRBS and eight for WGBS) to test DMR calling for various data configurations. Each subset has ten replicates. We downloaded them from http://www.bioinf.uni-leipzig.de/Software/metilene/Downloads/.

#### WGBS data from postnatal day 15 iron deficient and iron sufficient rat hippocampi

WGBS data were generated to analyze changes in hippocampal DNA methylation due to iron-deficiency during the fetal and neonatal periods. Pregnant/nursing rat dams were fed an iron-deficient diet (4 ppm iron) from gestational day 2 through postnatal day (P) 7, at which time they were given an iron-sufficient control diet (200 ppm iron). Iron sufficient control rats were fed an iron sufficient diet through the entire experimental duration. At P15, rat pups from both groups were euthanized and hippocampi were isolated. This diet manipulation induced a 60% iron deficiency in the P15 rat hippocampus [41, 42]. Three biological replicates of WGBS data were generated from hippocampi of both groups.

### Performance evaluation

We evaluate the DMR-identification programs Defiant, Metilene, MethylKit, MethylSig, RadMeth, and RnBeads. We chose not to use BSmooth and MOABS as they have already shown inferior performance as compared to Metilene [27] using the same test set we use in this experiment. Using the DMRs in the artificial datasets as the gold standard, we defined true positive (TP) when a predicted DMR overlapped with a DMR in the benchmark, otherwise it was defined as a false positive (FP). False negative (FN) was defined when a DMR in the benchmark dataset was not predicted. FN, FP, and TP values for all DMR callers for each artificial dataset are listed in Additional file 1: Table S1.

**Fig. 3:**
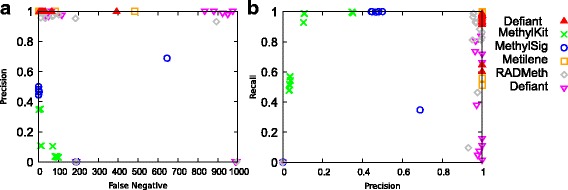
Performance comparison using all 16 Metilene’s benchmark datasets composed of RRBS and WGBS. Each point represents a data set. Precision is compared against FN (a) and recall against precision (b). In plot a, 15 points from Defiant, 15 from Metilene, 10 from RADMeth, and 5 from RnBeads overlap in the upper-left corner. Both Defiant and Metilene outperformed other predictors

For comprehensive evaluation, we compared precision (TP/(TP + FP)) against FN as well as against recall (TP/(TP + FN)) for 8 sets of RRBS and WGBS data (Fig. 3). In these tests, both Defiant and Metilene showed excellent precision and recall with very low FNs compared with other DMR callers. They scored perfect precision because their FP is 0 for all 16 tests. RnBeads also showed FP equal or close to zero. However, it suffered a high FN (Fig. 3). MethylKit showed the worst performance in these tests mainly due to excessive number of FPs. MethylSig showed high number of FPs for WGBS datasets (Additional file 1: Table S1). Moreover, MethylSig did not predict any DMRs for RRBS datasets (TPs and FPs were 0). RADMeth scored moderately well, but scored behind both Defiant and Metilene.

**Fig. 4:**
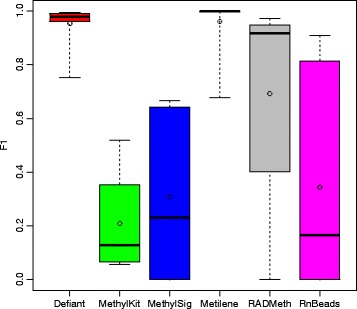
F1 values of 16 different artificial data sets. Circles indicate mean values. Both Defiant and Metilene outperformed other predictors

**Fig. 5:**
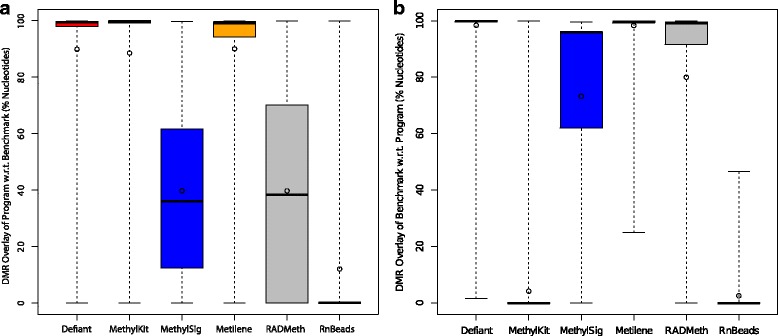
Comparison of DMR overlay of program with respect to benchmark in panel (**a**), and benchmark with respect to program in panel (**b**) for the combined RRBS and WGBS data sets

To obtain the overall performance we calculated the F1 score (=$2\times \frac{\mathrm{precision}\times \mathrm{recall}}{\mathrm{precision}+\mathrm{recall}}\Big)$. The F1 score is the harmonic mean of precision and recall. Both Defiant and Metilene showed outperforming F1 scores compared with other predictors (Fig. 4). The performances between Defiant and Metilene were comparable when we investigated the statistical differences between the predictions using a Welch’s t-test [24] (*p* = 0.81, Additional file 1: Table S2). Considering that the artificial datasets were generated using a beta binomial distribution, providing a favorable environment for Metilene, the comparable performance of Defiant is notable. Examining the results more closely, we found that Defiant outperformed Metilene especially for background 2, dataset 4 where there were subtle but significant differences in DNA methylation levels (Fig. 3 and Additional file 1: Table S1). To examine the DMR calling boundary more carefully, we calculated the overlapping ratios of DMRs, quantified as “DMR overlay” (Methods). Compared with Metilene, Defiant showed better overlay with respect to the predicted DMRs, suggesting that DMRs that Defiant calls capture the robust portion of DMRs (Fig. 5).

### Comparison using real WGBS data

We applied Defiant to identify DMRs in the iron-deficient rat hippocampus using the same parameters as were used with the artificial data. Fetal-neonatal iron deficiency, which is one of the most common nutritional deficiencies in the world [43], affecting as many as 2 billion people and approximately 30% of pregnancies [44, 45], causing long-term cognitive deficits despite iron treatment [46, 47]. Because these changes persist into adulthood even in the face of normal iron levels, and the known link between fetal exposures and long-term outcomes after birth, we investigated whether DNA methylation is altered in the developing rat hippocampus induced by fetal-neonatal iron-deficiency using WGBS datasets. The methods used to induce fetal-neonatal iron-deficiency have been previously described [48]. We investigated if DNA methylation is affected by iron-deficiency during fetal-neonatal periods using WGBS datasets with three iron-deficient rats and three iron sufficient rats.

**Fig. 6:**
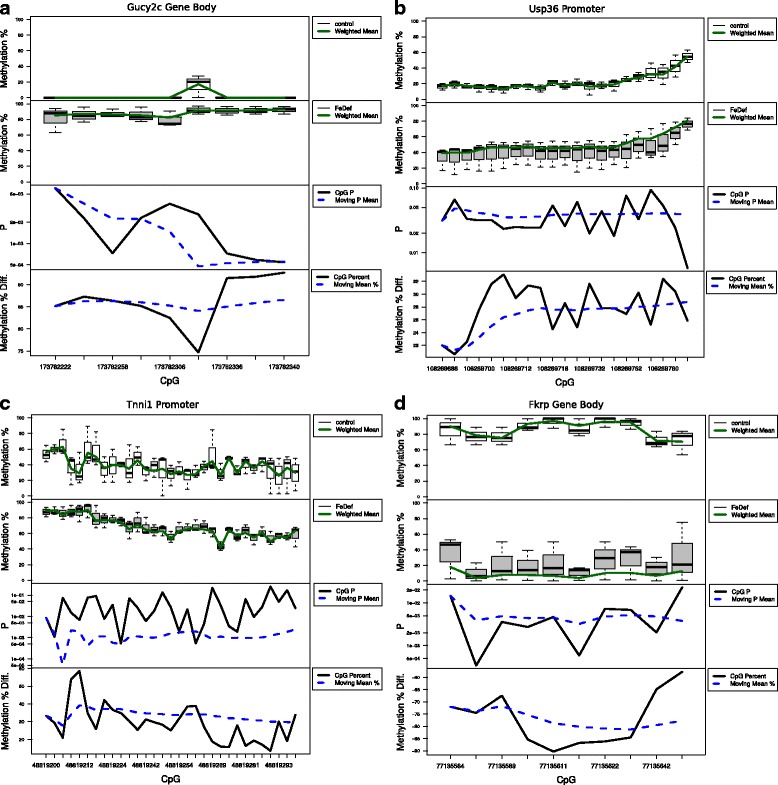
An iron-deficient diet induces strong changes in methylation for many genes, the strongest of which are shown here. **a** *Gucy2c* displays strongly increased methylation from an Fe-deficient diet. **b** *Usp36* is hypermethylated in Fe-deficient pups. **c** *Tnni1* shows moderate, but consistently significant hypermethylation. **d** *Fkrp* shows strong and consistent hypomethylation

We compared performance of Defiant to that of Metilene because they showed comparable performances based on the benchmarking test. Between the iron-deficient and iron-sufficient groups, Defiant identified 229 DMRs, while Metilene identified only 80 DMRs, with ten regions showing overlap between the two approaches. Figure 6 showed the DNA methylation status of the DMRs identified both by Defiant and Metilene.

**Fig. 7:**
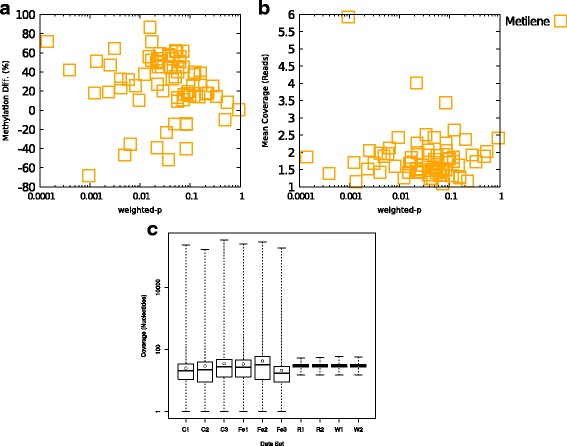
Statistics on CpG regions predicted uniquely by Metilene. While, these genes show differences in the methylation levels, their *p*-values were unacceptably high when coverage information is considered. **a** The differences in the methylation levels over weighted Welch’s *p*-values. **b** The mean coverage against the weighted *p*-value **c** distribution of coverage. Real WGBS shows much wider distribution of coverage than the artificial data set. “C” = iron sufficient rat samples, “Fe” = Iron-deficient rat samples, “R” = artificial RRBS data, and “W” = artificial WGBS data

**Fig. 8:**
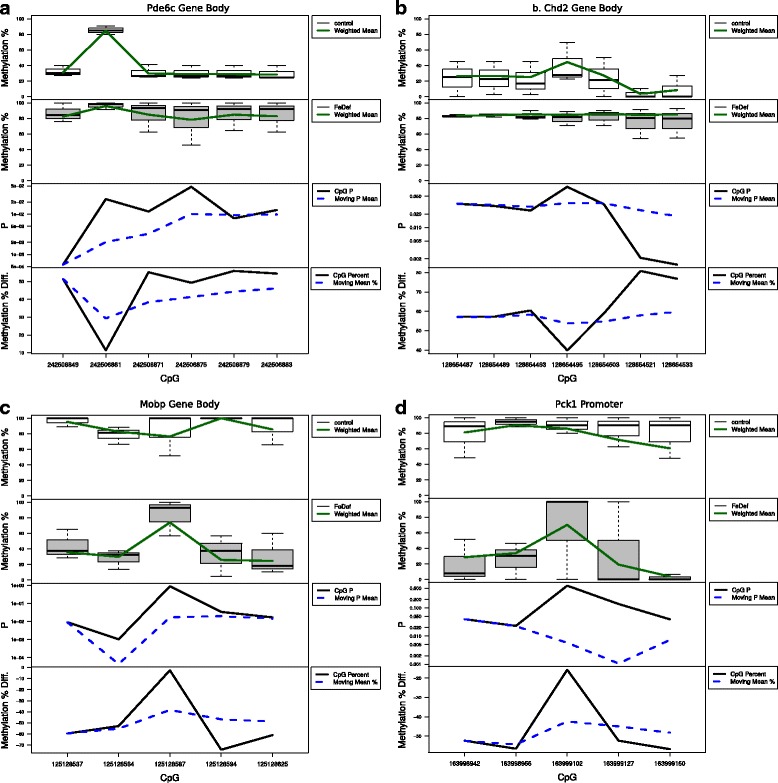
The DMRs identified by Defiant but missed by Metilene. DMRs on **a**. Pde6c gene body **b**. Cdh2 gene body, **c**. Mobp gene gody, and **d**. Pck1 promoter region

We found that the DMRs identified by Metilene but not by Defiant were mostly in low coverage areas. When methylation levels were weighted by the coverage, they showed non-significant *p*-values (Fig. 7a and b). Indeed, compared with the artificial datasets which have the distribution of coverage in a short range, the WGBS data in rat hippocampus were with a wider range of coverage (Fig. 7c). These results indicate a superior performance of Defiant when applied to real WGBS data. Figure 8 shows the examples of DMRs detected by Defiant but not by Metilene. The DNA methylation profiles showed clear differences in DNA methylation between the iron sufficient and the iron deficient groups for regions near genes such as *Pde6c, Chd2, Mobp*, and *Pck1*. The differences between Defiant’s and Metilene’s predictions are due to Defiant’s use of coverage-weighted means. The coverage-weighted means used in WWE allow Defiant to avoid artifacts such as Simpson’s paradox [49].

### Defiant identifies DMRs close to genes potentially affected by iron-deficiency in fetal-neonatal periods

The 229 DMRs that Defiant identified mapped within 15 Kbps of 108 genes (Additional file 1: Table S3). Among the 108 genes, 45 showed hypomethylated and 63 showed hypermethylated regions (Additional file 1: Tables S4 and S5, respectively). We identified that considerable portion of them (37 out of 43 hypomethylated and 31 out of 62 hypermethylated DMRs) were associated with neuronal function or development (Additional file 1: Tables S4 and S5), corroborating a previous finding [50]. Gene ontology (GO) analysis using Enrichr [51, 52] identified “abnormal nervous system” in Mouse Genome Informatics [53] mammalian phenotype level 3 (*p*-value = 0.005). Genes associated with this term were *Camk2b, Fkrp, Ncf1, St8sia1, Itsn1, Cacna1c, Usf2, Mib1, Fig4, Jph3, Mobp, Ush1g, Prkar1b, Tal1*, and *Pde6c*. We also observed terms “Rho GTPase cycle” (*p*-value: 0.0005), and “Axon guidance” (*p*-value = 0.001). In total, 7 out of 43 and 12 out of 62 genes were associated with GTPase activity for gain and loss of DNA methylation levels, respectively. The rho family of GTPases [54, 55], one of the G-protein coupled receptors, regulates neuronal morphogenesis [56], dendritogenesis [57], and spinogenesis [58]. We also found terms “synapse part” (*p*-value = 0.017; *Arf1, Tenm2, Pde2a, Cacna1c, Srgap2*, and *Mib1*). Collectively, Defiant identified DMRs close to the genes critical for neuronal synaptogenesis and plasticity.

### Defiant showed best performance based on the running time

Running time is important for genome-scale analysis. We evaluated the performance based on resource use. Using the benchmarking datasets, all pertinent DMR callers were run on the same system (Intel Xeon CPU 2.1GHz with 32GB RAM). For fair comparison, we converted the format of the benchmarking datasets into the format preferred by each DMR caller before evaluation. Therefore, mapping and input format conversion were not needed for this benchmarking. We observed dynamic range of running time and memory use. Defiant showed much faster performance compared with other methods: 5× faster than Metilene, 10× faster than MethylKit and MethylSig, and > 300× faster than RADMeth and RnBeads. When we applied Defiant to our WGBS rat datasets on the same system, it took less than 2 min to obtain the DMR results for the entire genome. Defiant’s memory usage is light, about 1GB for genome-wide WGBS data. Compared to the memory usages of the DMR callers, Defiant used slightly more memory than Metilene because Defiant is designed to run in a single step.

## Conclusions

We developed Defiant a new method to identify DMRs. Defiant is designed to provide easy and fast implementation of DMR calling while guaranteeing the prediction performance. We also put more credence to the CpGs with high coverage. For a rigorous test while weighing DNA methylation based on coverage, we used a Welch’s t-test. A Welch’s t-test does not assume equal variation between the sets and simplifies the incorporation of the weight information.

One of the widely used approaches for modeling DNA methylation is a beta binomial distribution. In our benchmarking tests, Defiant showed superior performance to other beta binomial distribution based predictors such MethylSig, MOABS, and RADMeth. It is noteworthy that the artificial data we used were generated by the developers of Metilene using a beta binomial distribution. Despite the potential bias against it, Defiant showed comparable performance with Metilene. Our results indicate that using a Welch’s t-test is appropriate for DMR identification. Close examination found that Defiant performed better then Metilene when modest but significant differences were observed (Fig. 3). More importantly, Defiant identified more DMRs in the rat hippocampus datasets which showed clear DMRs (Fig. 8). The DMRs uniquely observed by Metilene were in very low coverage areas (Fig. 7). Together, these suggest that Defiant performs better than Metilene for analysis of real data.

**Fig. 9:**
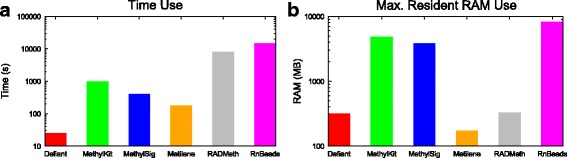
Resources used by DMR callers in time used (panel **a**) and RAM (panel **b**). We used eight artificial WGBS data sets. All evaluation was done on a computer with Intel(R) Xeon(R) CPU E5–2620 v2 @ 2.10GHz with 32 GB RAM

For genome-scale analysis, reduced running time is highly desirable. In our test, Defiant ran remarkably faster than other competitors using memory less than 1GB (Fig. 9). When applied to the whole-genome data in rats, it took less than 2 min for DMR calling for the entire genome. Defiant accepts diverse formats for DNA methylation including the format for Bismark coverage, and Bismark cytosine [59], BisSNP [60], MethylKit & MethylSig input, UCSC ENCODE, and EPP [61]. Defiant also runs as a standalone software.

For convenient analysis, Defiant provides annotation about the genes located around DMRs. Additional file 1: Table S3 shows the example of output of Defiant. Defiant is applicable to bisulfite-sequencing data, RRBS [19], HpaII tiny fragment enrichment by ligation-mediated PCR-tag (HELP-Tag) data [62], and Tet-assisted bisulfite sequencing (TAB-Seq) [63]. The source code is available on http://github.com/hhg7/defiant.

## Additional file

Additional file 1: Supplementary document. (PDF 475 kb)

## Abbreviations

DMR: Differentially methylated region
GO: Gene ontology
RRBS: Reduced Representation Bisulfite Sequencing
WGBS: Whole Genome Bisulfite Sequencing
WWE: Weighted Welch Expansion
