# Supplementary material for: Defiant: (DMRs: easy, fast, identification and ANnoTation) identifies differentially Methylated regions from iron-deficient rat hippocampus
Source: BMC Bioinformatics. 2018 Feb 5;19:31. doi: 10.1186/s12859-018-2037-1 (PMC5800085; doi:10.1186/s12859-018-2037-1)
Supplement: Additional file 1: — Supplementary document. (PDF 475 kb) [file 12859_2018_2037_MOESM1_ESM.pdf]

Supporting Information

David E. Condon, Phu V. Tran, Yu-Chin Lien, Jonathan Schug,
Michael K. Georgieff, Rebecca A. Simmons and Kyoung-Jae Won

Contents

S1 Artificial Data **2**

S2 DMR-Calling on Artificial Data Set **2**

S3 Rat Data **4**

S4 Effects of Parameters on Number of DMRs Identified in Rat Hippocampus **9**

S1 Artificial Data

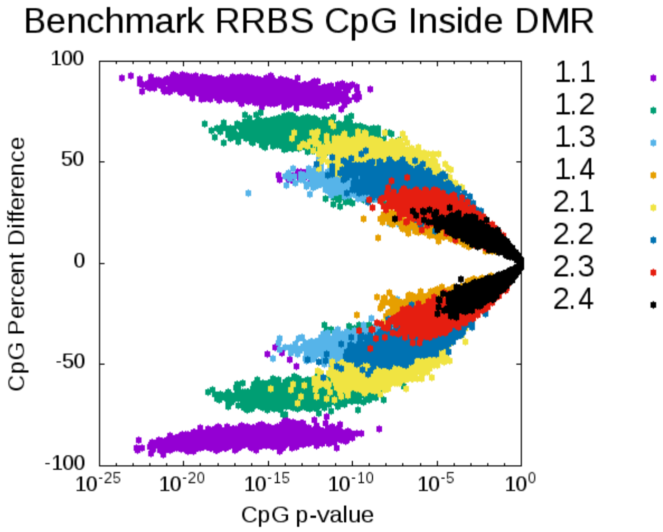

(a) CpGs within DMRs.

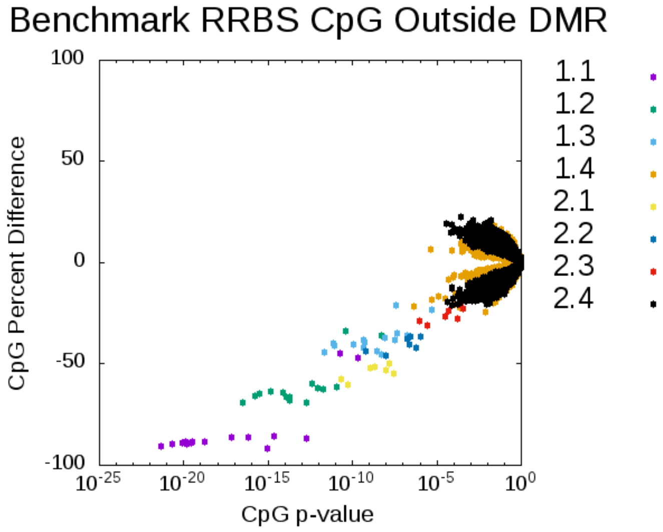

(b) CpGs outside of DMRs.

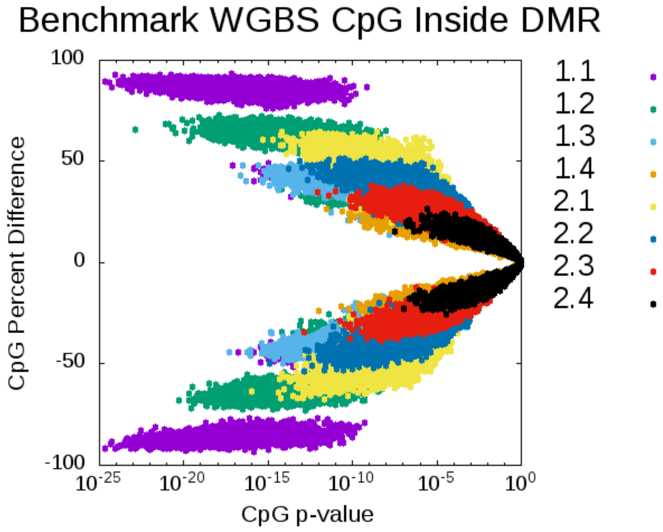

(c) CpGs within DMRs.

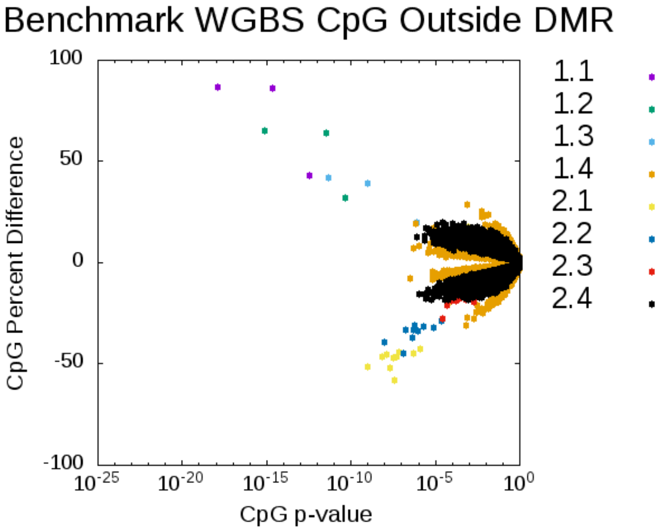

(d) CpGs outside of DMRs.

Figure S1: p-values and percent differences in the artificial data sets. Each point in each graph represents a single CpG. Background 1 DMRs show greater differences between groups than between group 2 sets. DMRs become increasingly similar to one another when progressing from sets 1-4 in both backgrounds, i.e. set 1.1 shows greater differences in methylation than set 1.2, etc.

S2 DMR-Calling on Artificial Data Set

	DMRs	Program	False Negative	False Positive	True Positive	Overlay	Reverse Overlay	Jaccard
**RRBS 1.1**	**191**	Defiant	4	0	187	97	99	0.97
		BH	4	0	187	97	99	0.97
		BY	4	0	187	97	99	0.97
		Holm	4	0	187	97	99	0.97
		Hochberg	4	0	187	97	99	0.97
		Bonferroni	4	0	187	97	99	0.97
**RRBS 1.2**	**191**	Defiant	12	0	179	93	99	0.93
		BH	10	0	181	94	99	0.94
		BY	10	0	181	94	99	0.94
		Holm	10	0	181	94	99	0.94
		Hochberg	10	0	181	94	99	0.94
		Bonferroni	10	0	181	94	99	0.94
**RRBS 1.3**	**191**	Defiant	14	0	177	92	99	0.92
		BH	12	0	179	93	99	0.93
		BY	12	0	179	93	99	0.93
		Holm	12	0	179	92	99	0.9
		Hochberg	12	0	179	92	99	0.9
		Bonferroni	12	0	179	92	99	0.9
**RRBS 1.4**	**191**	Defiant	12	0	179	91	99	0.91
		BH	8	0	183	92	98	0.79
		BY	5	0	186	89	94	0.51
		Holm	153	0	38	9	91	0.068
		Hochberg	153	0	38	9	91	0.068
		Bonferroni	153	0	38	9	91	0.068
**RRBS 2.1**	**188**	Defiant	9	0	179	94	93	0.57
		BH	9	0	179	95	99	0.93
		BY	9	0	179	95	99	0.93
		Holm	9	0	179	94	96	0.49
		Hochberg	9	0	179	94	96	0.49
		Bonferroni	9	0	179	94	96	0.49
**RRBS 2.2**	**188**	Defiant	14	0	174	92	93	0.56
		BH	11	0	177	94	99	0.92
		BY	10	0	178	95	98	0.85
		Holm	20	0	168	82	93	0.34
		Hochberg	20	0	168	82	93	0.34
		Bonferroni	20	0	168	81	93	0.33
**RRBS 2.3**	**188**	Defiant	16	0	172	90	93	0.55
		BH	13	0	175	93	94	0.46
		BY	15	0	173	85	93	0.38
		Holm	141	0	47	14	94	0.12
		Hochberg	141	0	47	14	94	0.12
		Bonferroni	141	0	47	14	94	0.12
**RRBS 2.4**	**188**	Defiant	66	0	122	50	96	0.39
		BH	186	0	2	0.24	95	0.0011
		BY	0	0	0	0	0	0
		Holm	0	0	0	0	0	0
		Hochberg	0	0	0	0	0	0
		Bonferroni	0	0	0	0	0	0
**WGBS 1.1**	**995**	Defiant	11	0	984	98	99	0.99
		BH	11	0	984	98	99	0.99
		BY	11	0	984	98	99	0.99
		Holm	11	0	984	98	99	0.98
		Hochberg	11	0	984	98	99	0.98
		Bonferroni	11	0	984	98	99	0.98
**WGBS 1.2**	**995**	Defiant	11	0	984	98	99	0.99
		BH	11	0	984	98	99	0.99
		BY	11	0	984	98	99	0.99
		Holm	11	0	984	1e+02	98	0.79
		Hochberg	11	0	984	1e+02	98	0.79
		Bonferroni	11	0	984	1e+02	98	0.79
**WGBS 1.3**	**995**	Defiant	19	0	976	97	99	0.98
		BH	19	0	976	98	99	0.98
		BY	17	0	978	99	98	0.86
		Holm	8	0	987	1.1e+02	88	0.37
		Hochberg	8	0	987	1.1e+02	88	0.37
		Bonferroni	8	0	987	1.1e+02	88	0.37
**WGBS 1.4**	**995**	Defiant	15	0	980	93	99	0.94
		BH	16	0	979	94	96	0.65
		BY	65	0	930	90	86	0.3
		Holm	943	0	52	2.8	78	0.025
		Hochberg	943	0	52	2.8	78	0.025
		Bonferroni	943	0	52	2.8	78	0.025
**WGBS 2.1**	**988**	Defiant	18	0	970	98	98	0.96
		BH	18	0	970	97	99	0.96
		BY	16	0	972	98	98	0.84
		Holm	98	0	890	90	86	0.32
		Hochberg	98	0	890	90	86	0.32
		Bonferroni	98	0	890	90	86	0.32
**WGBS 2.2**	**988**	Defiant	22	0	966	97	98	0.96
		BH	20	0	968	97	96	0.67
		BY	18	0	970	99	93	0.51
		Holm	350	0	638	57	83	0.23
		Hochberg	350	0	638	57	83	0.23
		Bonferroni	350	0	638	57	83	0.23
**WGBS 2.3**	**988**	Defiant	33	0	955	94	98	0.93
		BH	51	0	937	94	88	0.39
		BY	233	0	755	73	83	0.25
		Holm	920	0	68	4.4	82	0.039
		Hochberg	920	0	68	4.4	82	0.039
		Bonferroni	920	0	68	4.4	82	0.039
**WGBS 2.4**	**988**	Defiant	392	0	596	47	99	0.49
		BH	0	0	0	0	0	0
		BY	0	0	0	0	0	0
		Holm	0	0	0	0	0	0
		Hochberg	0	0	0	0	0	0
		Bonferroni	0	0	0	0	0	0

Table S1: Complete summary of data set performance. Each program is run on the artificial data set by Jühling, et al., and the performance is quantified according to column headings here. True negatives do not exist in this regime. Jüling, et al. made 200 DMRs in each RRBS data set, and 1000 DMRs in each WGBS data set. However, some of these DMRs overlap, which are combined to give the totals in the second column. This data is plotted in Figure 4.

	**Defiant**	**MethylKit**	**MethylSig**	**Metilene**	**RADMeth**	**RnBeads**
**Defiant**	1	6.8×-12	6.1×10^{-7}	0.81	0.02	1×10^{-5}
**MethylKit**	6.8×10^{-12}	1	0.3	1.1×10^{-12}	0.00024	0.22
**MethylSig**	6.1×10^{-7}	0.3	1	4×10^{-7}	0.0055	0.78
**Metilene**	0.81	1.1×10^{-12}	4×10^{-7}	1	0.018	8×10^{-6}
**RADMeth**	0.02	0.00024	0.0055	0.018	1	0.017
**RnBeads**	1×10^{-5}	0.22	0.78	8×10^{-6}	0.017	1

Table S2: Similarity of the results. A p-value is computed based on Welch's t-test to test if the F1 values are significantly different from each other. Each data set contains 16 F1 values, which are visualized in the main manuscripts Figure 3. Smaller p-values indicate two sets have similar prediction quality, and larger p-values indicate dissimilar prediction quality. Defiant and Metilene are very similar to each other, while MethylKit, MethylSig, and RnBeads are all similar.

S3 Rat Data

Table S3: Differentially Methylated Regions in rat hippocampus from iron-deficient diet. Percentage differences are weighted.

Chr	Start-End	CpN	ΔCpN	Mean.Diff.	Control	FeDef	p(DMR)	q(DMR, BH)	Gene
1	2984116-2984175	5	3	-54.1	68.2,87.8,94.1	35.5,3.5,66.7	0.030273	0.0370722	Ust
1	3283278-3283785	8	2	26.3	85.1,70.7,62.0	98.1,98.7,95.2	0.026841	0.0349238	n/a
1	3332817-3332836	5	4	49.0	47.1,30.0,32.4	99.2,79.0,41.3	0.0217799	0.0321781	n/a
1	14745012-14745130	5	2	-42.4	57.2,78.3,52.5	30.9,16.9,31.1	0.0112178	0.0254067	n/a
1	24003758-24003842	5	4	-52.9	63.5,97.3,85.9	18.9,25.4,62.7	0.0113219	0.0254067	n/a
1	41054728-41054799	5	4	-47.1	54.5,67.1,52.9	2.2,23.7,13.7	0.00374428	0.0176779	Tmem181
1	41178581-41178648	5	4	51.9	32.6,27.0,30.8	82.5,89.7,69.7	0.00405702	0.0178665	Ezr
1	53742719-53742971	5	2	50.5	68.4,31.1,43.8	98.7,97.2,82.0	0.0145519	0.0282406	n/a
1	54090073-54090645	5	2	11.8	73.5,61.0,58.8	73.1,74.5,86.3	0.0727801	0.074074	n/a
1	55893444-55893557	5	3	48.0	34.9,9.8,2.4	74.5,34.7,70.9	0.0181842	0.0307102	Riok2
1	77135564-77135647	10	10	-77.7	76.6,89.9,88.6	33.7,1.0,28.0	0.00378043	0.0176779	Fkrp
1	83975016-83975169	7	3	21.5	65.3,66.6,77.8	88.1,92.7,98.1	0.00861934	0.0241306	n/a
1	84451057-84451286	10	4	-20.6	35.2,43.6,23.4	5.2,19.9,10.0	0.0333063	0.0387164	Dpf1
1	85982567-85982780	10	4	-18.3	48.6,47.5,41.3	24.5,29.5,27.2	0.00256801	0.0162422	Usf2
1	87122965-87123053	6	3	55.1	47.7,1.4,4.6	53.4,89.9,74.6	0.0212011	0.0321527	Kctd15
1	111592052-111592130	5	5	34.7	29.1,32.2,30.8	66.5,66.4,64.3	2.32361e-06	0.000532106	Snurf
1	121044743-121044809	6	6	63.2	0.0,0.0,38.2	76.9,92.9,73.7	0.0155784	0.0291518	n/a
1	128654487-128654533	7	6	59.7	20.8,44.2,4.9	82.0,89.1,74.8	0.0178515	0.0307102	Chd2
1	158953236-158953343	5	3	28.6	46.6,35.1,42.3	62.1,80.0,64.6	0.0108184	0.0252358	Pde2a
1	171993800-171993818	8	6	73.6	1.0,1.0,100.0	100.0,55.0,100.0	0.0179782	0.0307102	Spon1
1	172182951-172183695	5	3	25.1	38.5,55.6,50.5	74.4,81.2,61.6	0.00926084	0.0241306	n/a
1	185363658-185363687	6	4	39.5	19.1,42.2,39.4	61.0,81.6,57.4	0.0116517	0.0255446	Sbk1
1	202868640-202869000	7	2	15.9	47.9,53.6,57.2	68.3,68.7,73.4	0.00932277	0.0241306	n/a
1	206461401-206461447	5	2	54.8	29.5,0.0,19.6	64.0,86.3,56.5	0.00531099	0.019938	Unc93b1
1	208319368-208319451	10	7	12.9	4.4,2.5,0.4	16.2,14.8,14.0	0.00144341	0.0162422	Map3k11
1	209815225-209815262	5	3	35.4	38.0,0.0,10.0	45.0,49.0,71.8	0.0236085	0.0329655	Macrod1
1	220326761-220326791	5	3	32.6	0.8,0.0,0.0	27.6,39.1,0.0	0.0327091	0.0385946	Foxb2
1	242506849-242506883	6	6	46.1	48.7,36.0,34.9	100.0,91.8,68.2	0.00979567	0.0242713	Pde6c
1	252626662-252626754	5	3	-39.4	38.3,56.3,48.6	11.0,4.7,16.9	0.00302388	0.0162422	Sh3pxd2a
1	267364979-267365031	7	5	12.2	0.0,0.0,0.0	13.6,5.2,11.8	0.0170925	0.0305796	n/a
10	4888901-4889304	9	6	-33.6	86.2,83.2,94.6	67.2,48.2,53.6	0.00524075	0.019938	n/a
10	11841326-11841423	5	2	-19.1	93.8,94.4,92.4	75.6,72.0,83.5	0.00872419	0.0241306	n/a
10	20351685-20351826	5	4	-41.5	73.3,73.2,85.2	45.2,26.6,60.2	0.0157616	0.0291518	Slit3
10	21254640-21254742	5	2	42.5	35.1,20.3,38.4	73.4,81.5,69.1	0.000983834	0.0162422	Tenm2
10	40271922-40271951	8	7	-25.1	19.2,30.4,29.0	0.6,0.0,2.5	0.0104426	0.0249099	Hint1
10	45545289-45545547	6	2	17.8	48.3,44.4,35.0	54.8,61.6,62.1	0.0117995	0.0255446	n/a
10	55714200-55714272	5	2	-41.6	25.9,60.9,40.8	5.4,0.0,29.2	0.0156958	0.0291518	Arhgef15
10	57112714-57112782	5	3	-40.9	40.0,57.7,53.7	23.5,7.6,11.0	0.00132764	0.0162422	n/a
10	67191436-67191780	9	3	25.2	76.8,51.7,59.7	84.8,95.6,73.5	0.0301709	0.0370722	n/a
10	77093819-77093868	5	5	71.5	10.8,16.7,10.3	93.9,78.0,75.9	0.00184672	0.0162422	n/a
10	105396059-105396099	9	6	-17.2	20.3,23.8,11.2	0.8,0.7,1.8	0.0282488	0.0365478	Ush1g
10	108269686-108269764	21	15	28.8	22.0,19.5,16.4	45.0,56.0,22.0	0.0391476	0.0431	Usp36
11	17146066-17146106	9	8	-30.2	42.6,53.0,59.0	35.0,7.4,24.7	0.0245269	0.0336327	n/a
11	31743497-31743549	5	4	29.8	47.7,46.7,27.5	68.7,71.7,61.4	0.0204757	0.0316667	Itsn1
11	63855759-63855814	5	3	36.8	5.7,0.0,6.9	58.1,38.7,21.5	0.0260422	0.0346725	Arhgap31
11	69580699-69580855	9	9	52.6	0.3,0.0,0.2	49.0,47.6,62.3	0.00525952	0.019938	Iqcg
12	10060115-10060156	5	3	-28.2	85.4,100.0,98.8	78.5,62.5,75.0	0.00706165	0.0231017	Trrap
12	16089265-16089348	5	4	40.2	0.0,18.6,8.5	58.6,49.8,27.5	0.00868512	0.0241306	Prkar1b
12	22788417-22788467	5	4	-36.6	69.9,82.4,88.6	23.2,53.3,49.0	0.0213802	0.0321556	Abhd11
12	23585373-23585418	9	7	-38.4	21.9,31.2,51.2	0.0,12.7,2.4	0.0221575	0.0322953	Ncf1
12	23791973-23792113	6	4	39.2	34.8,33.1,22.7	72.8,64.4,55.7	0.00124335	0.0162422	n/a
12	33719678-33719732	5	3	56.4	25.8,0.0,2.2	56.0,81.4,15.4	0.0254836	0.0343085	Hip1r
12	34621065-34621083	5	4	-37.7	39.4,55.7,65.6	12.8,3.7,28.8	0.00759221	0.0238167	Tmem120b
12	39062421-39062490	5	5	72.5	18.7,0.0,16.7	88.3,92.6,69.1	0.000822336	0.0162422	n/a
12	43111111-43111179	5	4	26.8	20.4,0.0,14.7	36.2,47.3,24.7	0.00996287	0.0242713	Ankrd13a
13	40660450-40660482	5	4	37.9	2.8,0.0,25.8	59.9,40.8,47.8	0.0139099	0.0276989	n/a
13	44269540-44269643	5	2	28.6	35.2,51.5,39.0	61.8,68.5,90.7	0.0128098	0.0259597	Srgap2
13	48819200-48819299	31	22	29.8	47.6,31.8,37.7	66.8,66.6,74.6	0.00247569	0.0162422	n/a
13	51913209-51913262	5	3	42.7	36.7,41.6,45.9	81.8,93.5,66.4	0.0186019	0.0307102	n/a
13	69053464-69053587	6	3	-34.2	53.3,79.4,55.3	18.2,39.0,43.0	0.0230969	0.0327881	n/a
13	94586882-94586912	6	6	-44.2	43.8,66.5,57.3	0.8,0.0,31.1	0.0184648	0.0307102	Kif26b
13	95168374-95168497	6	3	-28.2	69.9,87.0,88.6	38.0,69.3,90.2	0.110058	0.111519	Smyd3
13	106031408-106031457	5	3	30.1	62.6,54.4,56.6	86.0,90.1,79.5	0.00071932	0.0162422	Ptpn14
14	2958537-2958713	5	2	11.8	65.0,71.0,79.5	76.4,82.7,88.2	0.0288184	0.0368358	n/a
14	4804119-4804353	6	3	-38.3	66.5,65.2,100.0	45.7,36.6,66.0	0.03895	0.0431	n/a
14	9078808-9078864	6	2	-27.8	85.5,84.0,82.3	73.8,48.3,62.3	0.0437367	0.0472439	Cds1
14	11126297-11126452	8	6	42.1	4.9,1.0,0.0	40.8,52.8,34.3	0.00739733	0.0235276	n/a
14	49295945-49296146	5	3	10.6	58.6,65.0,64.4	72.2,78.5,67.0	0.0298388	0.0369357	n/a
14	78263930-78263981	5	4	30.9	19.5,0.4,5.2	34.2,42.8,36.2	0.0099039	0.0242713	n/a
14	83328062-83328105	7	4	37.4	20.5,0.0,0.5	53.0,15.8,43.5	0.0119357	0.0255446	Slc5a1
14	86532721-86532789	5	2	10.5	68.3,82.9,61.1	77.4,73.9,86.8	0.116593	0.11762	Aebp1
14	86683252-86683607	9	5	19.3	30.6,45.5,42.7	55.3,66.8,52.2	0.0216243	0.0321556	Camk2b
14	92399606-92399670	6	3	-35.1	74.2,86.6,81.8	50.5,40.8,61.1	0.00393701	0.0176779	n/a
14	103656026-103656093	6	4	-40.4	51.5,52.6,36.1	1.2,12.8,19.9	0.00284589	0.0162422	Commd1
15	18685274-18685409	7	3	27.3	11.4,1.1,1.1	24.4,31.2,36.5	0.0019221	0.0162422	Kctd6
15	47353634-47353650	5	5	35.4	2.1,0.0,9.7	0.0,46.2,29.1	0.0295973	0.0368358	n/a
15	56622270-56622384	5	2	27.4	61.6,65.9,59.5	94.1,93.0,68.9	0.020409	0.0316667	Cog3
15	57197525-57197705	5	3	46.7	54.2,35.4,46.4	88.2,99.0,84.5	0.00078294	0.0162422	n/a
15	71038760-71038830	7	4	-31.3	44.9,35.5,53.4	23.6,10.4,12.5	0.00478432	0.0192212	n/a
15	89916679-89916730	5	3	38.2	37.0,41.4,24.7	67.2,77.0,73.0	0.00283125	0.0162422	n/a
15	105901274-105901326	5	4	32.6	15.2,0.0,1.8	39.9,47.2,33.7	0.00470636	0.0192212	n/a
16	874927-874996	5	3	14.8	9.0,10.3,12.1	28.8,27.4,8.8	0.04543	0.0483882	n/a

Continued on next page

Table S3 – continued from previous page

Chr	Start-End	CpN	ΔCpN	Mean.Diff.	Control	FeDef	p(DMR)	q(DMR, BH)	Gene
16	2611707-2611912	6	5	-19.8	92.7,96.1,88.8	64.4,77.4,56.4	0.0453399	0.0483882	Arhgef3
16	9778982-9779163	6	2	-19.5	83.9,100.0,87.1	73.8,71.4,64.3	0.0284361	0.0365835	n/a
16	18886302-18886380	8	5	-20.4	29.7,22.0,20.0	2.1,0.9,2.9	0.00698341	0.0231017	Jak3
16	36318586-36318678	5	4	-34.3	42.9,58.3,41.0	18.3,7.9,25.0	0.00573369	0.0211776	n/a
16	60564878-60564937	7	4	-19.4	6.2,24.1,20.4	0.3,0.0,3.2	0.0179139	0.0307102	Ppp1r3b
16	76246820-76247048	6	3	33.4	7.0,21.7,10.3	44.5,51.3,31.6	0.0041934	0.0181187	n/a
16	76945869-76945907	6	6	-48.5	51.5,71.3,57.0	22.2,4.6,13.6	0.00201781	0.0162422	n/a
16	78707244-78707433	6	3	23.1	53.8,46.1,50.0	68.0,79.0,71.3	0.00494369	0.019519	n/a
16	83012122-83012301	7	2	17.4	20.7,6.1,16.6	30.8,33.4,33.6	0.0228205	0.0326618	n/a
17	4281441-4281478	8	8	33.7	0.0,1.7,18.9	37.1,54.2,1.8	0.0325863	0.0385946	n/a
17	8854702-8854734	5	5	15.9	0.0,0.3,0.0	19.0,17.2,13.2	0.00601435	0.0215201	n/a
17	15935669-15935742	5	4	32.7	41.7,39.7,57.0	83.1,86.7,76.6	0.0100801	0.0242983	n/a
17	22396243-22396561	7	2	19.6	75.1,78.7,69.8	95.6,92.0,88.4	0.00218851	0.0162422	n/a
17	22609252-22609299	5	4	-56.5	57.5,100.0,81.5	28.7,25.7,53.3	0.00784158	0.0241306	n/a
17	26064417-26064604	5	3	26.5	15.5,41.6,35.6	76.1,50.1,63.8	0.0319702	0.0385325	n/a
17	73067990-73068071	6	2	-19.7	90.8,93.4,79.5	67.1,64.8,83.2	0.0183347	0.0307102	Adarb2
17	81114169-81114311	7	3	15.2	74.3,81.9,73.5	90.6,89.5,92.3	0.00923972	0.0241306	n/a
17	93284212-93284310	8	4	43.1	15.7,0.0,12.6	65.1,61.3,34.0	0.0191771	0.0307102	Armc3
18	2156838-2156872	6	6	48.9	38.1,0.0,17.8	78.7,63.2,17.2	0.0202432	0.0316667	Mib1
18	23768658-23768682	9	9	22.3	0.4,0.0,12.9	19.1,32.5,18.6	0.0128081	0.0259597	n/a
18	24357111-24357179	10	8	-11.6	12.2,12.5,8.2	1.0,0.0,0.0	0.000746369	0.0162422	Lims2
18	31022806-31022976	5	2	-29.9	74.1,96.6,78.3	48.3,59.0,53.7	0.0189068	0.0307102	n/a
18	31178559-31178577	5	2	-17.2	30.3,26.2,42.1	13.2,8.3,17.7	0.020214	0.0316667	RGD735029
18	49143687-49143733	7	5	-33.0	35.0,0.0,43.6	4.2,0.3,0.8	0.0475656	0.0504283	n/a
18	54715910-54716038	5	4	58.5	24.9,38.7,15.2	86.9,95.8,69.3	0.00179423	0.0162422	Adamts19
18	63553630-63553823	9	3	-15.1	81.2,76.0,77.9	58.3,68.6,62.7	0.0132092	0.0265342	n/a
19	14099548-14099609	5	3	-22.4	90.7,100.0,96.9	79.2,73.1,73.2	0.00293713	0.0162422	Rasd2
19	22720661-22720747	6	2	24.3	64.5,74.8,56.6	80.2,94.9,95.6	0.0175453	0.0307102	n/a
19	22849465-22849522	6	3	-39.0	96.9,100.0,87.5	72.4,44.5,76.0	0.0291378	0.0368358	Dnaja2
19	25001054-25001122	6	5	37.6	42.5,17.8,50.4	94.7,69.5,69.1	0.0231951	0.0327881	n/a
19	25712441-25712473	5	5	-37.6	29.7,48.8,39.1	0.0,0.5,3.5	0.00923059	0.0241306	n/a
19	38713521-38713538	5	2	46.9	33.3,10.9,38.1	69.2,82.5,59.3	0.0086472	0.0241306	n/a
19	38980387-38980420	5	4	-23.1	70.0,80.0,88.4	65.3,59.7,53.8	0.016757	0.0304455	n/a
19	52002579-52002798	8	2	25.1	69.0,76.9,60.7	97.4,91.4,85.7	0.00908934	0.0241306	Jph3
19	52899132-52899183	5	2	33.3	57.8,64.3,50.4	95.1,86.3,81.6	0.00130456	0.0162422	Pabpn1l
2	12283994-12284047	5	3	27.9	2.0,0.0,0.0	39.0,28.4,13.5	0.0157852	0.0291518	n/a
2	29168336-29168379	9	4	21.2	2.1,0.0,0.3	0.0,28.2,17.5	0.0495389	0.0518009	n/a
2	31424242-31424271	6	2	17.9	0.0,0.0,3.1	20.7,11.4,17.6	0.00430524	0.0182159	Marveld2
2	121891228-121891476	6	3	-40.8	64.5,75.1,67.4	25.9,37.5,22.6	0.000798562	0.0162422	n/a
2	181597557-181597692	5	4	-58.8	76.3,95.5,90.2	35.2,13.6,27.7	0.00124966	0.0162422	n/a
2	221045909-221045998	5	3	21.1	73.8,73.9,78.5	98.5,97.0,88.4	0.00220147	0.0162422	n/a
2	235588411-235588653	7	4	-43.3	56.8,93.1,72.8	42.6,21.7,41.8	0.0125395	0.0259597	n/a
2	242429702-242430218	5	2	-23.4	73.1,81.0,86.4	54.7,52.9,68.3	0.00922422	0.0241306	n/a
2	244585404-244585449	6	3	59.7	45.9,82.4,17.9	97.0,100.0,63.0	0.0328644	0.0385946	n/a
20	8714262-8714300	5	5	23.6	0.0,0.9,1.2	34.1,18.6,18.2	0.0322619	0.0385946	Btbd9
20	10508413-10508515	6	2	-20.5	91.5,95.2,93.4	68.8,75.3,71.4	0.00238295	0.0162422	n/a
20	12719388-12719472	10	10	-57.3	63.1,11.1,72.6	7.9,5.0,3.4	0.033603	0.0388641	Dip2a
20	14232267-14232470	7	4	22.5	31.2,20.1,50.0	69.0,58.6,49.6	0.0622895	0.0636799	n/a
20	21840729-21840811	17	14	31.1	58.3,61.6,41.0	63.0,89.3,89.8	0.025619	0.0343085	n/a
20	43959021-43959029	5	5	16.7	64.1,66.3,71.0	83.9,83.0,82.8	0.00664756	0.0227207	Rev3l
20	45220690-45220716	5	5	47.9	15.3,0.0,17.7	52.1,69.9,61.1	0.00150237	0.0162422	Fig4
3	512284-513118	6	2	12.5	69.3,62.3,72.3	83.5,80.7,79.5	0.0222823	0.0322953	n/a
3	857533-857571	6	3	-21.5	90.4,83.3,85.7	67.5,62.8,75.9	0.00391654	0.0176779	n/a
3	6093327-6093452	5	3	-42.9	45.2,72.0,62.0	21.7,5.1,25.6	0.00598267	0.0215201	Sardh
3	8484968-8485016	6	5	-37.6	34.0,60.6,48.2	29.1,0.0,19.2	0.018827	0.0307102	n/a
3	23226986-23227030	11	9	37.4	57.1,45.9,33.6	77.7,87.2,56.4	0.0190621	0.0307102	n/a
3	52516532-52516792	5	2	17.1	85.2,85.3,79.2	100.0,99.6,100.0	0.00956842	0.0242094	Myo3b
3	90642611-90642715	5	2	-45.6	66.2,100.0,98.9	47.1,12.3,76.1	0.0409278	0.0448444	LOC691083
3	114681988-114682283	6	2	-22.4	59.3,44.0,64.4	50.2,30.2,34.3	0.0295529	0.0368358	n/a
3	142445820-142445854	6	4	36.8	14.8,8.2,4.1	38.1,61.0,32.0	0.0313729	0.0380126	Scrt2
3	142623641-142623664	6	6	13.5	0.0,0.0,0.0	16.1,14.1,0.0	0.0376158	0.0420196	Tbc1d20
3	145186118-145186253	5	3	47.6	18.4,20.9,21.5	58.9,86.5,53.8	0.03125	0.0380126	n/a
3	147228964-147229028	5	2	32.2	73.2,61.3,61.3	97.1,94.2,94.1	0.00317907	0.0165456	n/a
3	147652273-147652381	5	4	55.5	47.7,0.0,23.6	72.5,86.9,84.7	0.0202811	0.0316667	n/a
3	163145309-163145362	5	4	42.2	39.5,28.1,47.5	97.0,83.5,75.8	0.00219583	0.0162422	n/a
3	163998942-163999150	5	3	-48.2	81.0,89.2,65.8	27.5,27.0,38.9	0.00724292	0.023361	n/a
3	169107440-169107470	6	5	-18.6	17.7,23.2,0.0	0.2,2.0,0.9	0.0369772	0.0415087	Ss18l1
4	18887802-18887867	5	3	-36.5	93.3,82.7,91.5	54.9,45.0,84.6	0.0294021	0.0368358	n/a
4	30043178-30043287	6	3	-10.0	96.0,98.4,93.1	85.5,86.4,86.0	0.0150873	0.0287916	Pon2
4	64617299-64617342	5	3	51.3	71.6,28.5,46.7	90.8,100.0,80.6	0.0126591	0.0259597	Dgki
4	64694008-64694079	5	3	35.6	0.6,0.0,0.0	34.3,42.9,19.2	0.0215731	0.0321556	n/a
4	79078961-79078979	5	5	-36.5	83.3,100.0,72.7	35.9,53.8,57.1	0.0161661	0.0296162	n/a
4	83224864-83224882	6	2	-23.3	16.6,45.8,35.5	15.4,5.1,26.5	0.0492341	0.0517184	n/a
4	116220692-116221039	6	3	23.9	58.8,65.2,88.5	86.1,91.9,96.3	0.0576563	0.0592075	Mrpl19
4	117883970-117884074	5	2	-27.7	79.1,83.6,91.4	72.4,49.5,66.1	0.0233906	0.0328616	n/a
4	125493483-125493628	7	5	35.4	65.2,52.4,57.8	97.3,98.6,65.0	0.0348407	0.0396494	n/a
4	135068059-135068209	6	3	25.6	13.8,0.6,12.2	44.5,34.2,23.1	0.0109894	0.0252358	n/a
4	135419854-135420078	5	4	29.5	28.6,26.4,4.8	66.9,32.8,46.5	0.0501873	0.0522404	n/a
4	152175717-152175986	5	2	37.3	49.6,53.9,38.5	82.0,92.9,70.9	0.00389964	0.0176779	n/a
4	155004956-155005063	5	2	27.9	28.9,0.0,11.6	40.5,33.3,55.8	0.0219386	0.0322048	Cacna1c
4	156652330-156652470	5	4	31.8	70.2,40.5,50.4	82.7,80.8,89.0	0.0349401	0.0396494	B4galnt3
4	173782222-173782340	9	9	86.6	2.2,2.9,0.0	93.6,88.5,80.6	0.000555303	0.0162422	Gucy2c
4	180853644-180853654	6	2	-20.1	23.8,21.8,22.8	5.2,0.0,2.6	0.00293218	0.0162422	St8sia1
4	183227074-183227255	5	2	29.4	54.7,48.2,43.5	83.5,73.9,79.0	0.00134202	0.0162422	n/a
5	23291095-23291135	11	9	26.5	77.6,68.4,57.4	96.4,83.8,96.2	0.0189427	0.0307102	Clvs1
5	36423056-36423067	5	4	24.5	0.0,2.0,0.3	22.8,30.5,17.2	0.0183721	0.0307102	n/a
5	47480035-47480257	7	3	-20.7	21.0,25.0,25.6	4.4,3.2,2.4	0.000921858	0.0162422	n/a
5	74610996-74611190	5	2	-17.4	15.3,20.2,24.5	2.3,1.2,14.2	0.00704929	0.0231017	n/a
5	122024952-122025056	6	2	-35.3	74.2,55.1,73.9	20.9,18.7,40.3	0.0123664	0.0259597	n/a
5	122084769-122084873	6	4	56.0	16.7,0.0,21.0	68.0,80.1,63.4	0.000769255	0.0162422	Ak4
5	135420990-135421016	7	6	-35.1	56.7,67.3,62.2	22.5,18.8,40.3	0.00915786	0.0241306	n/a
5	136132686-136132721	5	4	-56.8	57.4,87.2,74.1	47.1,7.9,3.2	0.01102	0.0252358	Mknk1
5	136160604-136160687	7	3	19.8	3.1,0.0,1.2	19.5,23.6,20.3	0.000397944	0.0162422	n/a
5	149435589-149435615	5	4	-52.3	78.9,90.2,100.0	40.0,45.7,28.9	0.00103301	0.0162422	n/a
6	12391051-12391125	15	12	24.9	39.3,44.6,44.7	71.5,59.8,77.5	0.0168846	0.0304455	Ppp1r21
6	55087640-55087713	5	3	-37.4	48.0,46.8,47.4	0.5,10.1,28.3	0.0209088	0.0319208	n/a
6	73701796-73701933	5	2	40.6	57.0,45.5,42.7	84.3,93.3,79.7	0.00115759	0.0162422	n/a
6	96289551-96289692	6	3	60.8	47.2,12.2,33.1	90.3,90.9,68.4	0.00622269	0.021923	n/a
6	107440678-107440705	6	3	-13.3	10.1,16.0,19.1	0.3,6.0,0.5	0.00377919	0.0176779	Dpf3
6	122268714-122268843	8	5	28.1	10.0,6.4,1.7	47.7,31.6,27.2	0.00903122	0.0241306	n/a
6	128589455-128589471	5	5	11.9	77.3,75.0,79.6	89.7,92.0,83.9	0.00266009	0.0162422	n/a
6	132317229-132317284	6	4	44.7	15.8,32.0,16.6	59.8,73.3,30.8	0.0265253	0.0347103	Bcl11b
6	137232424-137232529	7	4	29.7	30.5,45.0,45.0	65.4,74.7,66.2	0.0036151	0.0176779	n/a
7	5989545-5989557	5	2	21.1	72.8,60.0,75.0	94.0,96.8,90.4	0.00304985	0.0162422	n/a
7	12927921-12927963	5	3	-45.3	86.0,80.0,87.2	23.4,42.7,73.4	0.0248557	0.0338807	Wiz
7	16313904-16313927	5	3	28.7	2.0,0.4,3.4	37.7,24.5,22.1	0.0173605	0.0307102	Kank3
7	20973985-20974033	6	4	42.0	25.0,9.1,26.2	52.0,79.9,68.8	0.0114275	0.0254067	Ric8b
7	21538395-21538476	7	3	33.7	10.9,0.0,2.2	24.0,54.4,42.1	0.0411516	0.0448748	n/a
7	32468442-32468577	5	2	38.7	27.5,17.2,0.7	39.6,72.5,64.9	0.0225117	0.0324225	n/a
7	48108685-48110000	8	3	-14.8	96.9,90.4,81.9	72.7,81.6,64.3	0.050689	0.0525239	n/a
7	58578533-58578764	5	3	26.6	68.5,41.5,55.4	82.6,89.5,82.3	0.0326632	0.0385946	n/a
7	71564289-71564366	6	3	45.6	31.9,19.9,19.8	83.1,62.5,70.2	0.00286265	0.0162422	n/a
7	112895011-112895202	5	3	20.5	59.0,56.8,40.1	73.9,60.2,66.7	0.0349084	0.0396494	n/a
7	125015154-125015274	6	2	-38.5	54.6,58.8,57.6	29.2,6.4,33.8	0.0294466	0.0368358	n/a
7	126341158-126341239	5	3	-28.3	30.8,34.1,31.9	8.7,0.9,5.2	0.00257108	0.0162422	n/a
7	135265350-135265404	5	3	38.2	33.3,19.1,4.3	61.8,50.8,54.1	0.0148276	0.0285338	Slc38a1
7	139805770-139805786	5	2	14.6	3.8,5.4,1.4	10.6,21.9,8.0	0.0426613	0.0463007	n/a
7	142086661-142086804	5	4	60.8	30.5,5.5,23.5	82.1,99.5,60.2	0.00882773	0.0241306	n/a

Continued on next page

Table S3 – continued from previous page

Chr	Start-End	CpN	ΔCpN	Mean.Diff.	Control	FeDef	p(DMR)	q(DMR, BH)	Gene
8	6090401-6090446	6	4	28.4	45.3,67.3,57.6	89.0,83.3,83.9	0.0245255	0.0336327	n/a
8	12721284-12721410	9	7	50.1	16.7,5.4,0.0	72.3,51.1,47.5	0.004375	0.0182159	Fat3
8	41254229-41254287	6	3	30.8	49.2,37.1,46.8	70.5,78.4,63.8	0.00230177	0.0162422	n/a
8	51154948-51155149	5	2	-18.3	50.3,55.6,52.3	15.2,50.8,34.1	0.186609	0.186609	n/a
8	53698160-53698290	5	2	38.9	40.6,37.0,39.7	82.0,73.1,82.4	0.00239481	0.0162422	n/a
8	65166756-65166792	6	6	28.1	71.2,63.6,72.9	98.8,99.6,97.4	0.00276201	0.0162422	n/a
8	91817444-91817491	5	4	-69.0	83.4,100.0,58.3	6.9,14.8,17.2	0.00962034	0.0242094	Pgm3
8	104424662-104424727	9	4	-10.7	34.9,32.0,25.3	21.4,18.6,18.7	0.0367213	0.0414245	Armc8
8	107270544-107270587	5	3	33.2	16.4,0.0,0.0	47.5,43.9,7.9	0.0349745	0.0396494	Ephb1
8	108640761-108640829	7	5	16.8	65.7,72.2,72.5	93.2,85.7,84.8	0.00282423	0.0162422	n/a
8	125128537-125128625	5	4	-48.9	85.0,84.5,94.8	55.1,28.6,55.4	0.0144395	0.0282406	Mobp
8	127158930-127158988	5	2	-25.0	47.0,29.9,28.6	0.0,15.2,10.0	0.0140888	0.0278133	Klhl40
9	9045295-9045389	6	3	-29.0	61.6,67.1,58.9	26.1,48.7,38.4	0.0206041	0.0316667	Guca1a
9	10483790-10483920	6	2	18.8	70.2,54.4,61.1	83.5,79.7,77.6	0.02386	0.0331148	n/a
9	11436153-11436327	6	3	-44.0	82.9,85.7,88.1	52.0,32.5,45.3	0.00937828	0.0241306	n/a
9	18057850-18057912	5	3	22.6	3.2,0.0,0.0	29.1,19.5,5.1	0.0263564	0.0346874	Tfap2b
9	31431836-31431978	6	2	24.2	40.7,24.7,33.2	50.8,62.6,48.1	0.0106525	0.0251486	n/a
9	32492545-32492748	6	2	-21.8	70.5,76.3,94.4	48.0,62.6,74.6	0.0567747	0.0585649	Bag2
9	74595534-74595562	5	3	-14.3	0.0,14.0,18.4	0.4,0.0,1.6	0.0481507	0.0508134	Dnpep
9	77468091-77468349	5	2	10.0	76.3,88.7,71.8	91.7,89.5,89.1	0.141672	0.142293	n/a
9	77904490-77904556	5	3	27.4	59.1,49.9,49.1	65.4,88.1,78.4	0.0263288	0.0346874	Mogat1
9	91559815-91560325	5	3	36.2	53.0,42.4,32.8	77.1,75.9,72.7	0.0119306	0.0255446	n/a
9	107124722-107125470	5	2	-21.2	88.5,76.7,87.2	64.8,55.7,71.8	0.0122189	0.0259086	Arhgap28
X	90842868-90842894	6	6	31.4	33.2,21.1,40.5	50.0,71.2,65.4	0.00652632	0.0226444	n/a
X	90843046-90843140	12	9	32.4	33.3,20.3,23.1	76.3,48.5,51.2	0.0331123	0.0386873	n/a
X	122513987-122514019	6	3	28.8	69.0,57.4,70.0	96.6,96.1,94.0	0.00850166	0.0241306	n/a
X	130298607-130299446	7	2	10.8	59.1,61.6,52.3	61.9,72.9,65.0	0.0447212	0.0480806	n/a
X	160446080-160446146	5	5	-55.2	59.0,97.1,93.8	31.4,34.9,28.1	0.0250428	0.0339338	n/a
X	160539343-160539409	6	3	-38.2	78.5,100.0,74.9	64.9,30.9,65.2	0.0391317	0.0431	n/a

Gene	# CpG	ΔMethylation	Description
Fkrp†[1]	10	-77.7	fukutin related protein
Dip2a†[2]	10	-57.3	disco interacting protein 2 homolog A
Dpf1†[3,4]	10	-20.6	D4, zinc and double PHD fingers family 1
Usf2†[5]	10	-18.3	upstream transcription factor 2
Lims2†[6,7]	10	-11.6	LIM and senescent cell antigen like domains 2
Ncf1†[8]	9	-38.4	neutrophil cytosolic factor 1
Ush1g	9	-17.2	Usher syndrome 1G
Armc8†[9]	9	-10.7	armadillo repeat containing 8
Hint1†[10]	8	-25.1	histidine triad nucleotide binding protein 1
Jak3*†[11]	8	-20.4	Janus kinase 3
Ppp1r3b*	7	-19.4	protein phosphatase 1, regulatory (inhibitor) subunit 3B
Tal1†[12]	7	-35.1	T cell acute lymphocytic leukemia 1
Arhgef3*†[13]	6	-19.8	Rho guanine nucleotide exchange factor (GEF) 3
Bag2†[14]	6	-21.8	BCL2-associated athanogene 2
Ss18l1†[15]	6	-18.6	synovial sarcoma translocation gene on chromosome 18-like 1
Adarb2†[16]	6	-19.7	adenosine deaminase, RNA-specific, B2
Kif26b†[17]	6	-44.2	kinesin family member 26B
Guca1a*†[18]	6	-29.0	guanylate cyclase activator 1a (retina)
Cds1	6	-27.8	CDP-diacylglycerol synthase 1
Dnaja2†[19]	6	-39.0	DnaJ heat shock protein family (Hsp40) member A2
Commd1†[20]	6	-40.4	COMM domain containing 1
St8sia1†[21]	6	-20.1	ST8 alpha-N-acetyl-neuraminide alpha-2,8-sialyltransferase 1
Smyd3	6	-28.2	SET and MYND domain containing 3
Dpf3†[4]	6	-13.3	D4, zinc and double PHD fingers, family 3
Pon2†[22]	6	-10.0	paraoxonase 2
Pgm3*†[23]	5	-69.0	phosphoglucomutase 3
Arhgap28*†[24]	5	-21.2	Rho GTPase activating protein 28
Mknk1*†[25]	5	-56.8	MAP kinase-interacting serine/threonine kinase 1
LOC691083	5	-45.6	Unknown
Slit3†[26]	5	-41.5	slit homolog 3 (Drosophila)
Tmem120b†[27]	5	-37.7	transmembrane protein 120B
Klhl40†[28]	5	-25.0	kelch-like 40
Pck1*†[29]	5	-48.2	phosphoenolpyruvate carboxykinase 1, cytosolic
Dnpep†[30]	5	-14.3	aspartyl aminopeptidase
Mobp†[31]	5	-48.9	myelin-associated oligodendrocytic basic protein
RGD735029	5	-17.2	Unknown
Trrap†[32]	5	-28.2	transformation/transcription domain-associated protein
Tmem181†[33]	5	-47.1	Unknown
Sardh†[34]	5	-42.9	sarcosine dehydrogenase
Wiz†[35]	5	-45.3	widely-interspaced zinc finger motifs
Ust†[36]	5	-54.1	uronyl-2-sulfotransferase
Arhgef15†[37]	5	-41.6	Rho guanine nucleotide exchange factor (GEF) 15
Sh3pxd2a†[38]	5	-39.4	SH3 and PX domains 2A

Table S4: DMRs showing decreased methylation from an Fe-deficient diet in rat hippocampus data. DMRs are associated with genes based on a promoter cutoff of 15,000 nucleotides. Gene names are from MGI.

*Has role in GTPases and nucleotide exchange.

†Is associated with the hippocampus, neurons, and/or brain, with relevant literature cited.

Gene	# CpG	ΔMethylation	Description
Tnni1†[39]	31	29.8	troponin I, skeletal, slow 1
Usp36†[40]	21	28.8	ubiquitin specific peptidase 36
Ppp1r21/Ccdc128*[41]	15	24.9	protein phosphatase 1, regulatory subunit 21
Clvs1	11	26.5	clavesin 1
Map3k11*†[42]	10	12.9	mitogen-activated protein kinase kinase kinase 11
Camk2b†[43]	9	19.3	calcium/calmodulin-dependent protein kinase II, beta
Iqcg†[44]	9	52.6	IQ motif containing G
Gucy2c*†[45]	9	86.6	guanylate cyclase 2c
Armc3†[46]	8	43.1	armadillo repeat containing 3
Spon1†[47]	8	73.6	spondin 1, (f-spondin) extracellular matrix protein
Jph3†[48]	8	25.1	junctophilin 3
Slc5a1†[49]	7	37.4	solute carrier family 5 (sodium/glucose cotransporter), member 1
Kctd6†[50]	7	27.3	potassium channel tetramerisation domain containing 6
Chd2†[51]	7	59.7	chromodomain helicase DNA binding protein 2
Capn12†[52]	7	21.5	calpain 12
Scrt2†[53]	6	36.8	scratch homolog 2, zinc finger protein (Drosophila)
Mrpl19†[54]	6	23.9	mitochondrial ribosomal protein L19
Tbc1d20†[55]	6	13.5	TBC1 domain family, member 20
Ak4*†[56]	6	56.0	adenylate kinase 4
Kctd15†[57]	6	55.1	potassium channel tetramerisation domain containing 15
Bcl11b†[4]	6	44.7	B cell leukemia/lymphoma 11B
Mib1†[58]	6	48.9	mindbomb homolog 1 (Drosophila)
Sbk1*†[59]	6	39.5	SH3-binding kinase 1
Pde6c*†[60]	6	46.1	phosphodiesterase 6C, cGMP specific, cone, alpha prime
Marveld2†	6	17.9	MARVEL (membrane-associating) domain containing 2
Ric8b†[61]	6	42.0	resistance to inhibitors of cholinesterase 8 homolog B (C. elegans)
Arf1†[62]	6	17.8	ADP-ribosylation factor 1
Myo3b†[63]	5	17.1	myosin IIIB
Dgki*†[64]	5	51.3	diacylglycerol kinase, iota
Unc93b1	5	54.8	unc-93 homolog B1 (C. elegans)
Rev3l	5	16.7	REV3-like, catalytic subunit of DNA polymerase zeta RAD54 like (S. cerevisiae)
Foxb2	5	32.6	forkhead box B2
Olr987	5	21.1	Unknown
Prkar1b*†[65]	5	40.2	protein kinase, cAMP dependent regulatory, type I beta
Btbd9†[66]	5	23.6	BTB (POZ) domain containing 9
Riok2*	5	48.0	RIO kinase 2 (yeast)
Pde2a†[67]	5	28.6	phosphodiesterase 2A, cGMP-stimulated
Itsn1†[68]	5	29.8	intersectin 1 (SH3 domain protein 1A)
Mc3r	5	42.2	melanocortin 3 receptor
Ptpn14*	5	30.1	protein tyrosine phosphatase, non-receptor type 14
B4galnt3	5	31.8	beta-1,4-N-acetyl-galactosaminyl transferase 3
Fat3	5	62.8	FAT atypical cadherin 3
Kank3	5	28.7	KN motif and ankyrin repeat domains 3
Ankrd13a	5	26.8	ankyrin repeat domain 13a
Tfap2b	5	22.6	transcription factor AP-2 beta
Cog3	5	27.4	component of oligomeric golgi complex 3
Macrod1	5	35.4	MACRO domain containing 1
Ezr	5	51.9	ezrin
Arhgap31*	5	36.8	Rho GTPase activating protein 31
Mogat1	5	27.4	monoacylglycerol O-acyltransferase 1
Srgap2*	5	28.6	SLIT-ROBO Rho GTPase activating protein 2
Tenm2	5	42.5	teneurin transmembrane protein 2
Pabpn1l	5	33.3	poly(A)binding protein nuclear 1-like
Slc38a1	5	38.2	solute carrier family 38, member 1
Tgif2	5	32.2	TGFB-induced factor homeobox 2
Hip1r	5	56.4	huntingtin interacting protein 1 related
Cacna1c	5	27.9	calcium channel, voltage-dependent, L type, alpha 1C subunit
Aebp1	5	10.5	AE binding protein 1
Fig4*	5	47.9	FIG4 phosphoinositide 5-phosphatase
Snurf	5	34.7	SNRPN upstream reading frame
Adamts19	5	58.5	a disintegrin-like and metallopeptidase (reprolysin type) with thrombospondin type 1 motif, 19
Ephb1	5	33.2	Eph receptor B1

Table S5: DMRs showing increased methylation from rat hippocampus data. Gene names are from MGI. Has role in GTPases and nucleotide exchange. ‡is associated with calmodulin in Hematopoiesis

S4 Effects of Parameters on Number of DMRs Identified in Rat Hippocampus

Option	Default value when not specified
**minimum coverage**	10
**difference of methylation percentage**	10.000000
**p-value**	0.05
**minimum number of CpN**	5
**minimmm range of the differentially methylated nucleotides**	0
**maximum range between CpN**	20,000
**non-differentially methylated CpG count for extension**	5

Table S6: Parameters used for evaluating individual and combinined parameters. All images in this section show two parameters varied as all others are set to default values.

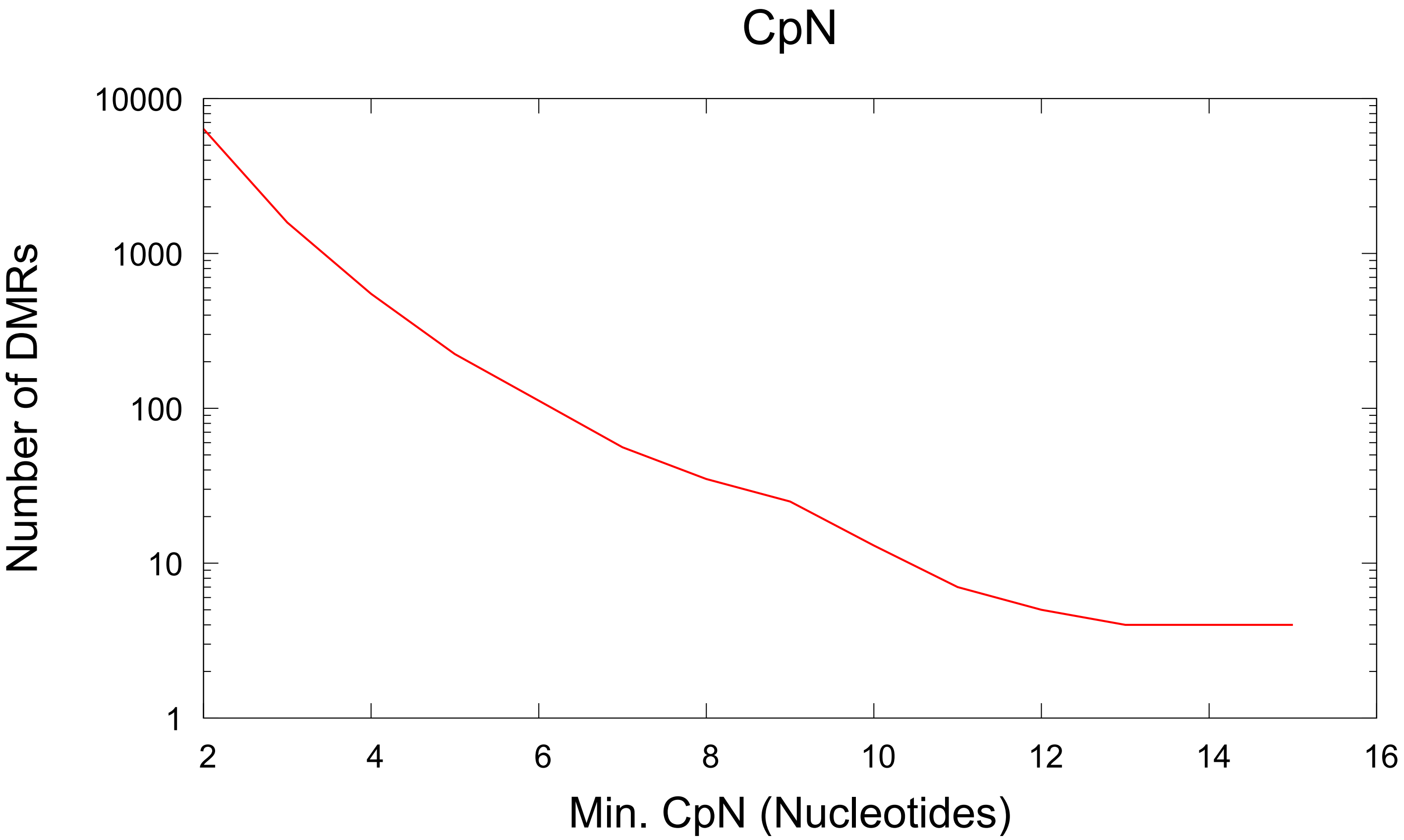

Figure S2: DMRs counted as a function of minimum CpN, with all other options held constant as indicated in Table S6. This plot is made with GNUPlot using the Perl script plot_results.pl which is included with Defiant. Lower minimum CpN counts show more and often shorter DMRs.

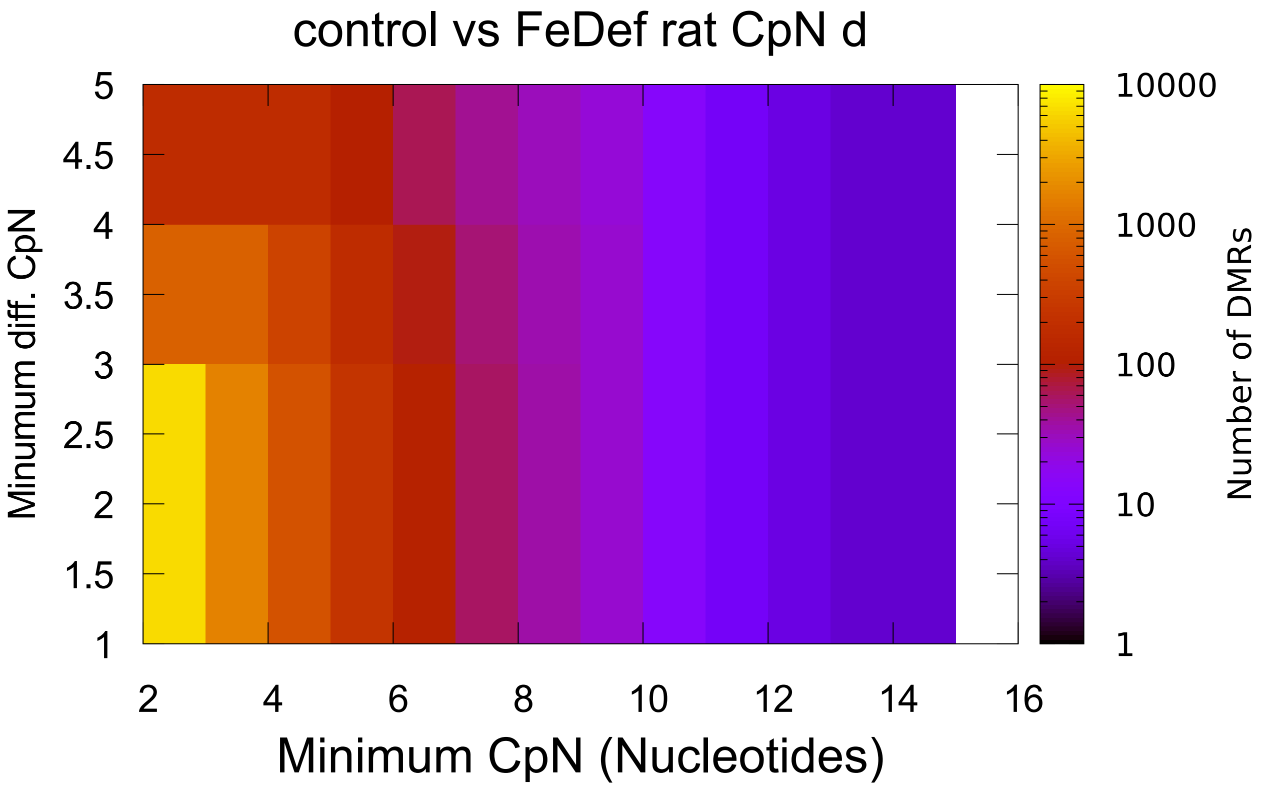

Figure S3: DMRs in rat hippocampus counted as a function of CpN and differentially methylated CpN (d), with all other parameters held constant as indicated in Table S6. This plot is made with GNUPlot using the Perl script plot_results.pl which is included with Defiant. Similar sharp decreases in DMR ount occur as in Figure S2.

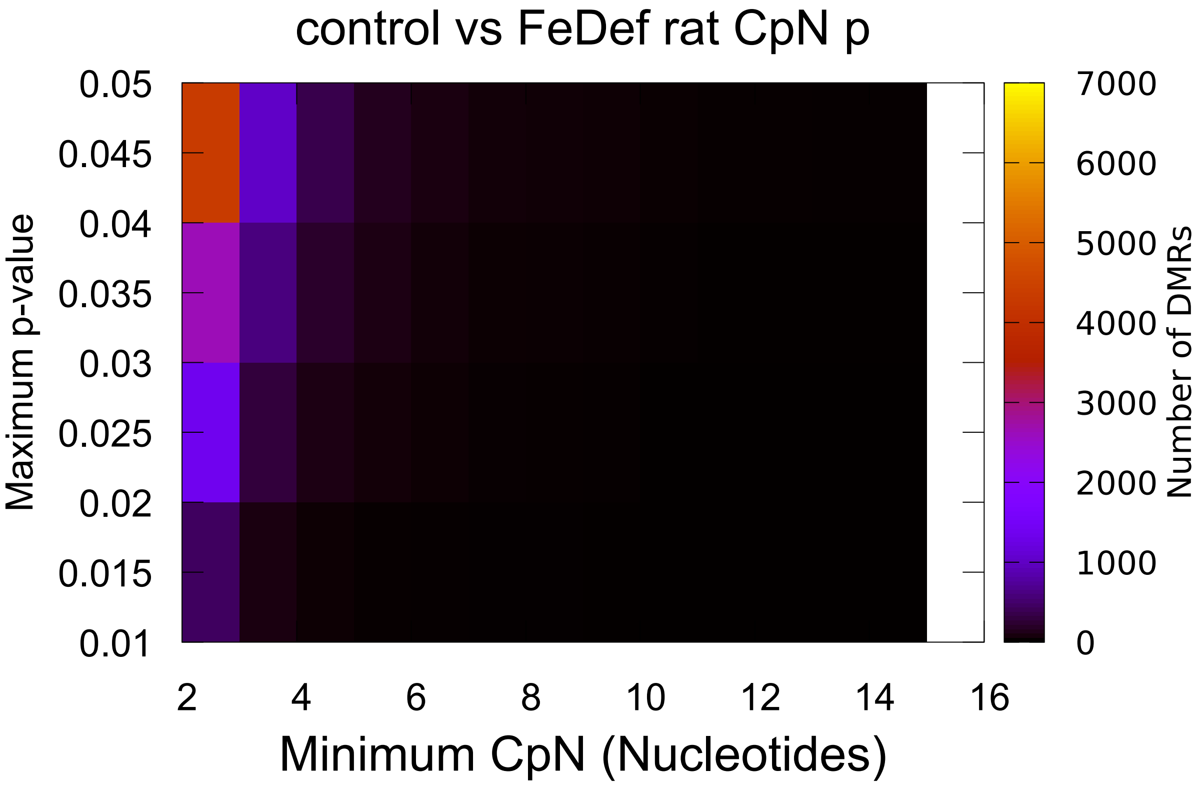

Figure S4: DMRs in rat hippocampus counted as a function of CpN and p-value with all other parameters held constant as indicated in Table S6. This plot is made with GNUPlot using the Perl script plot_results.pl which is included with Defiant. Low p-value and low CpN will give a high DMR count, but many are likely false positives.

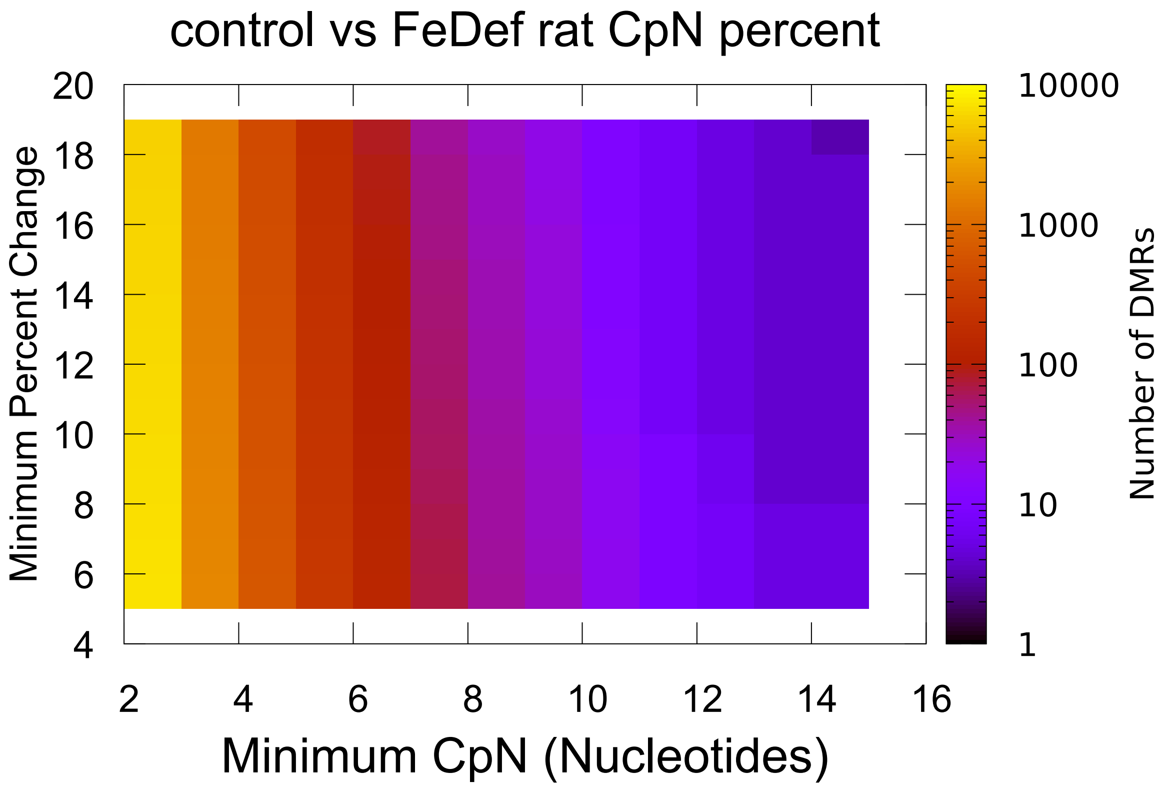

Figure S5: DMRs in rat hippocampus counted as a function of CpN & minimum percent cutoff. All other parameters held constant as indicated in Table S6. This plot is made with GNUPlot using the Perl script plot_results.pl which is included with Defiant. CpN appears to be more important than minimum percent change.

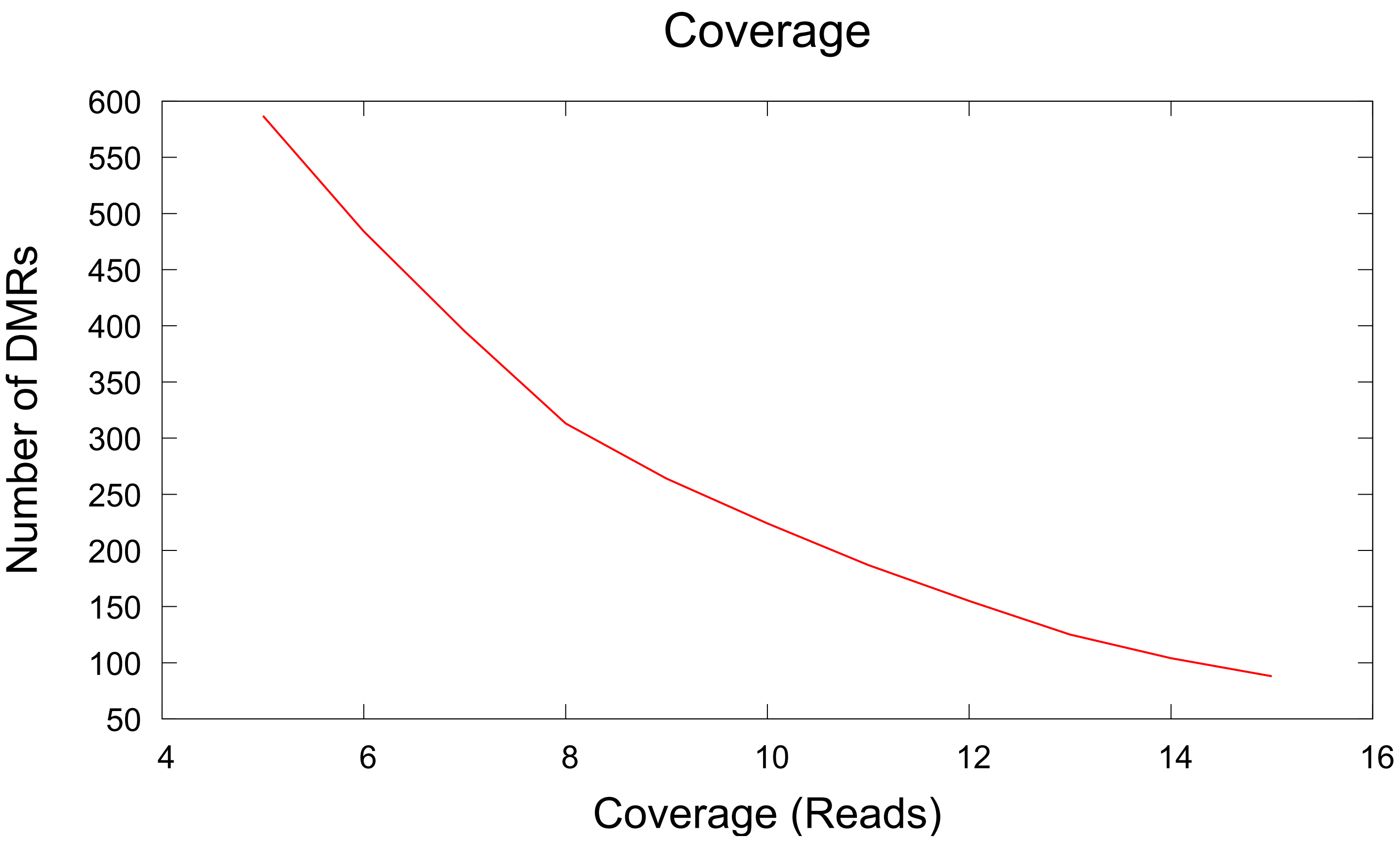

Figure S6: Minimum coverage influences the number of DMRs found in the rat hippocampus with all other parameters set constant as in Table S6. This plot is made with GNUPlot using the Perl script plot_results.pl which is included with Defiant. Low coverage has higher sensitivity, but poorer selectivity, while high coverage will have better sensitivity, but poorer selectivity.

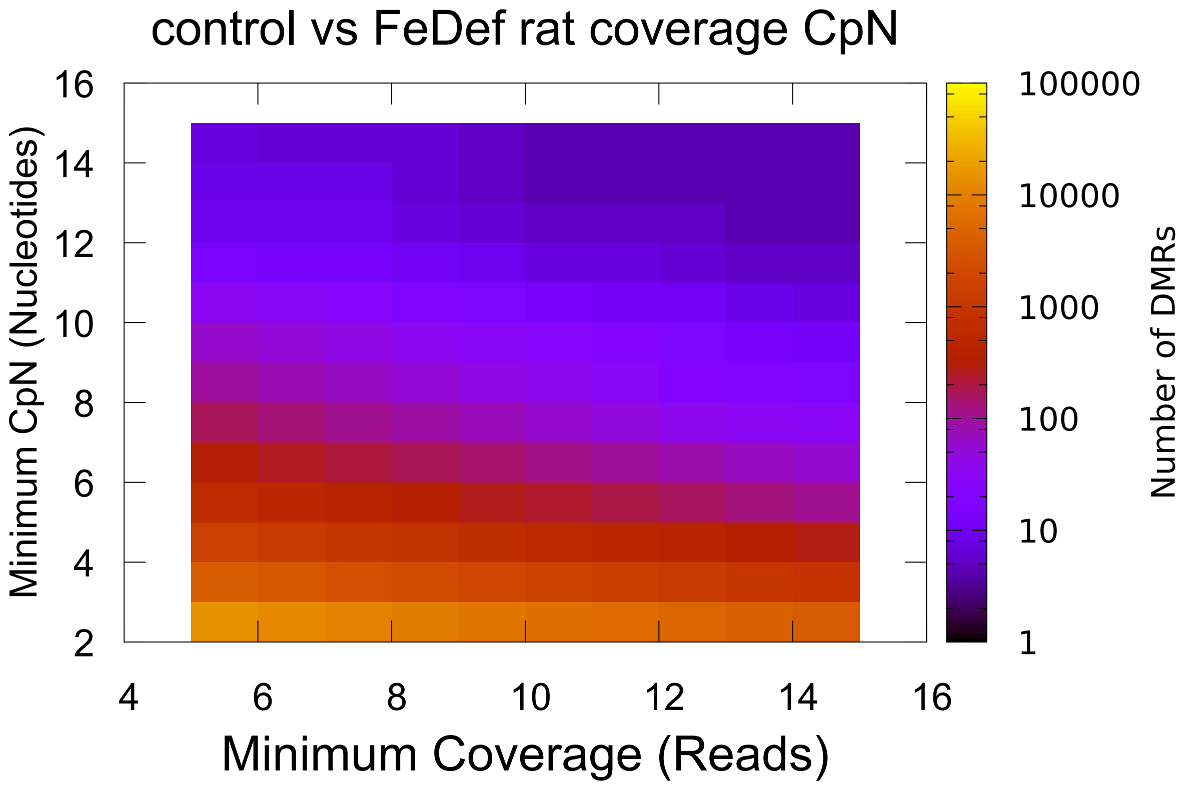

Figure S7: A sample graph showing number of DMRs found in the rat hippocampus as a function of minimum coverage and minimum CpN. All parameters but coverage and CpN are set as in Table S6. This plot is made with GNUPlot using the Perl script plot_results.pl which is included with Defiant. Both exponentially increase the number of DMRs, which necessitates a logarithmic scale.

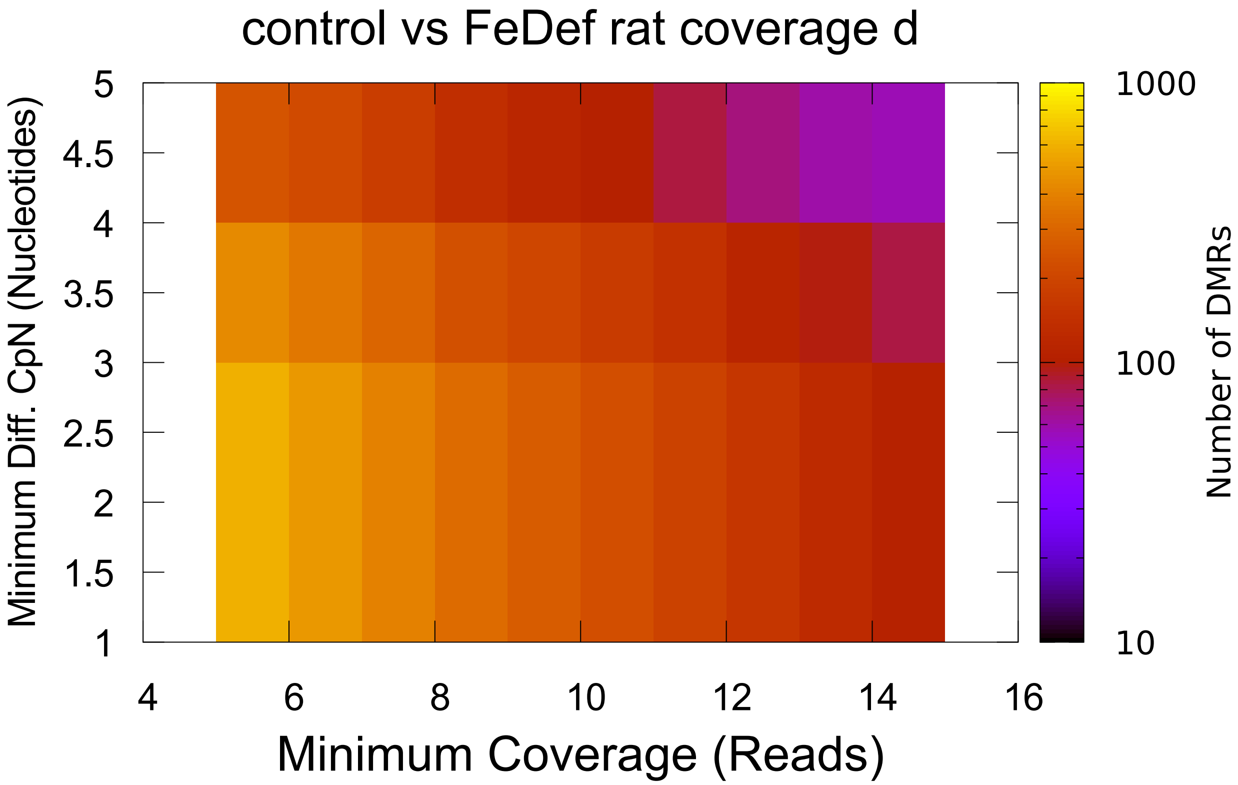

Figure S8: Minimum coverage and minimum differential CpN work similarly together to determine the DMR count in the rat hippocampus. All other parameters held constant as indicated in Table S6. This plot is made with GNUPlot using the Perl script plot_results.pl which is included with Defiant.

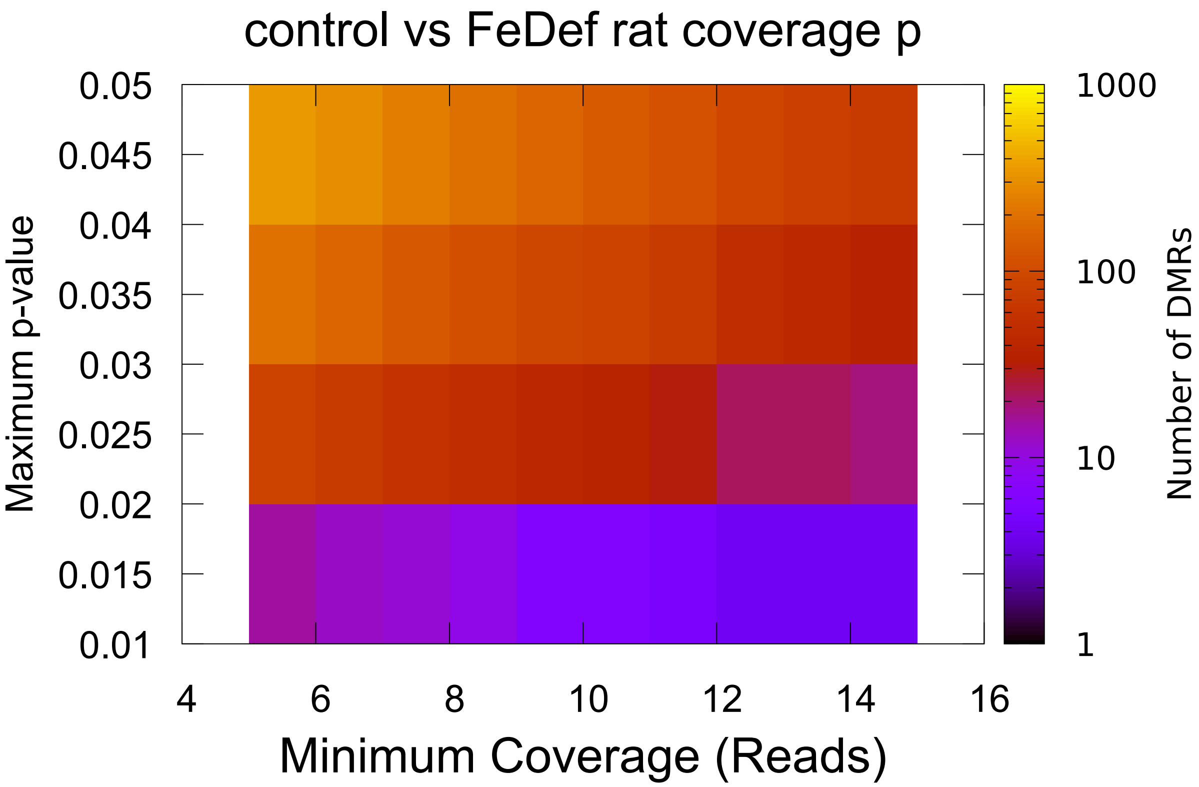

Figure S9: Minimum coverage and p value influence the number of DMRs found in the rat hippocampus. All other parameters held constant as indicated in Table S6. This plot is made with GNUPlot using the Perl script plot_results.pl which is included with Defiant. P-value appears slightly more important than coverage in determining DMR count.

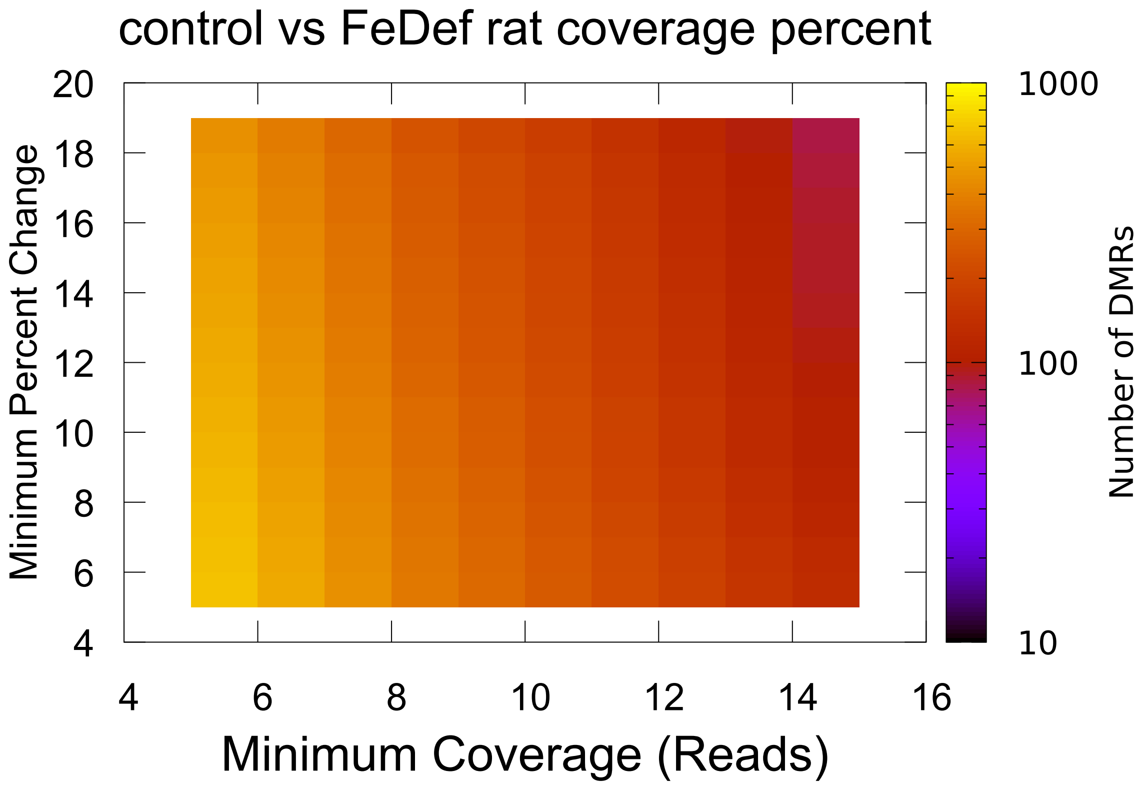

Figure S10: The number of DMRs found in the rat hippocampus is influenced by the minimum coverage and the minimum percent cutoff. All other parameters held constant as indicated in Table S6. This plot is made with GNUPlot using the Perl script plot_results.pl which is included with Defiant.

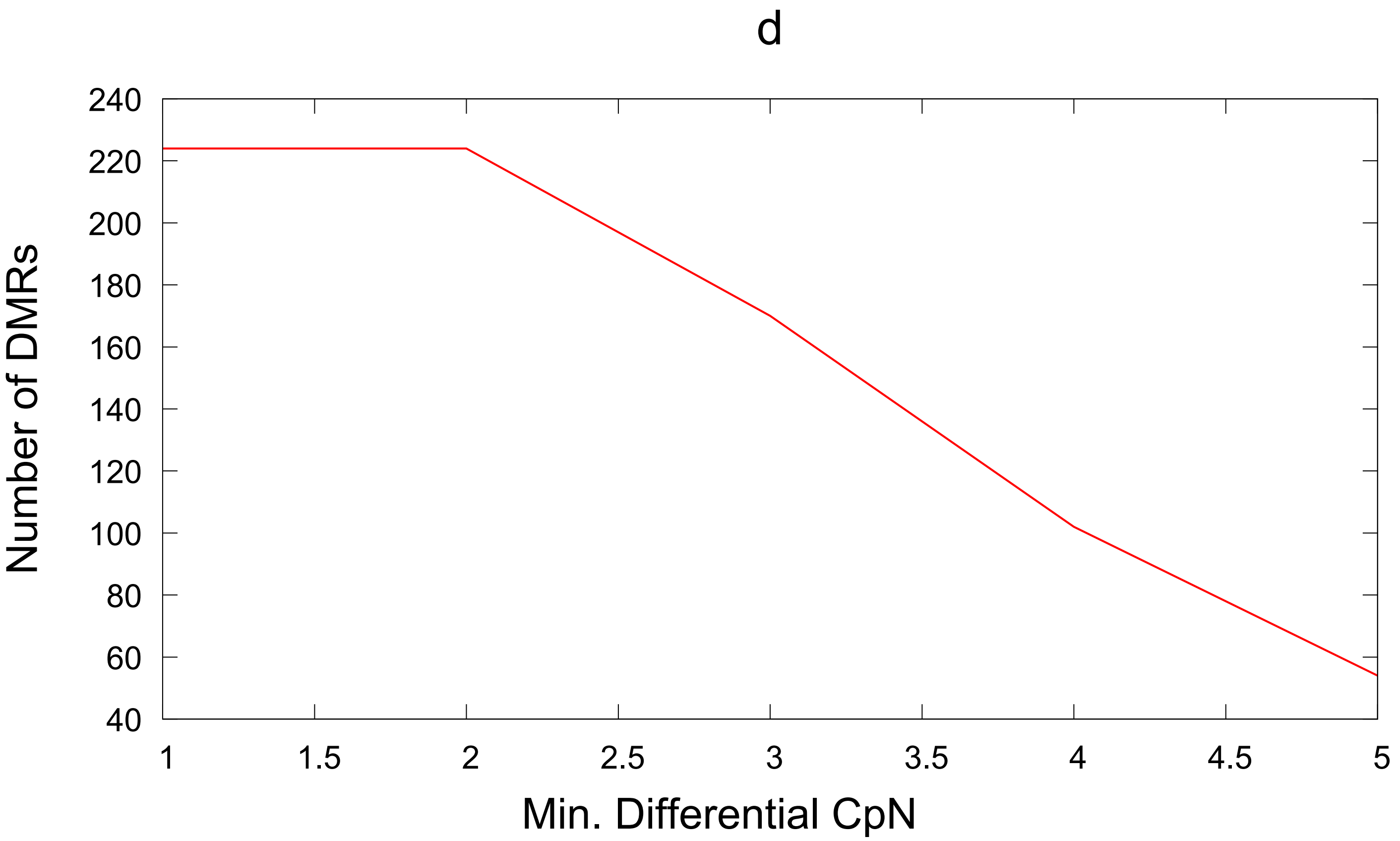

Figure S11: How the minimum number of differentially methylated CpN influences the number of DMRs in the rat data. All other parameters are held constant as in Table S6. This plot is made with GNUPlot using the Perl script plot_results.pl which is included with Defiant.

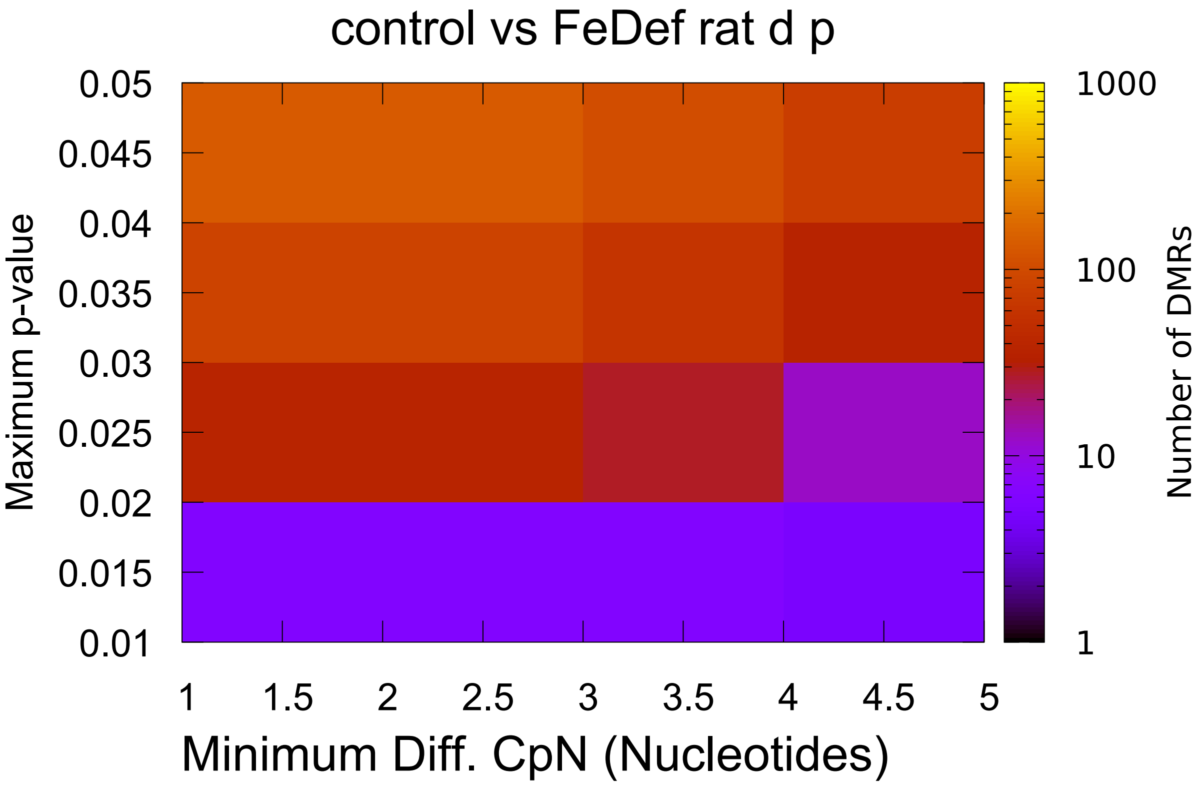

Figure S12: The minimum number of differentially methylated CpN "d" and the maximum p-value influence the number of DMRs found in the rat hippocampus. All other parameters held constant as indicated in Table S6. This plot is made with GNUPlot using the Perl script plot_results.pl which is included with Defiant.

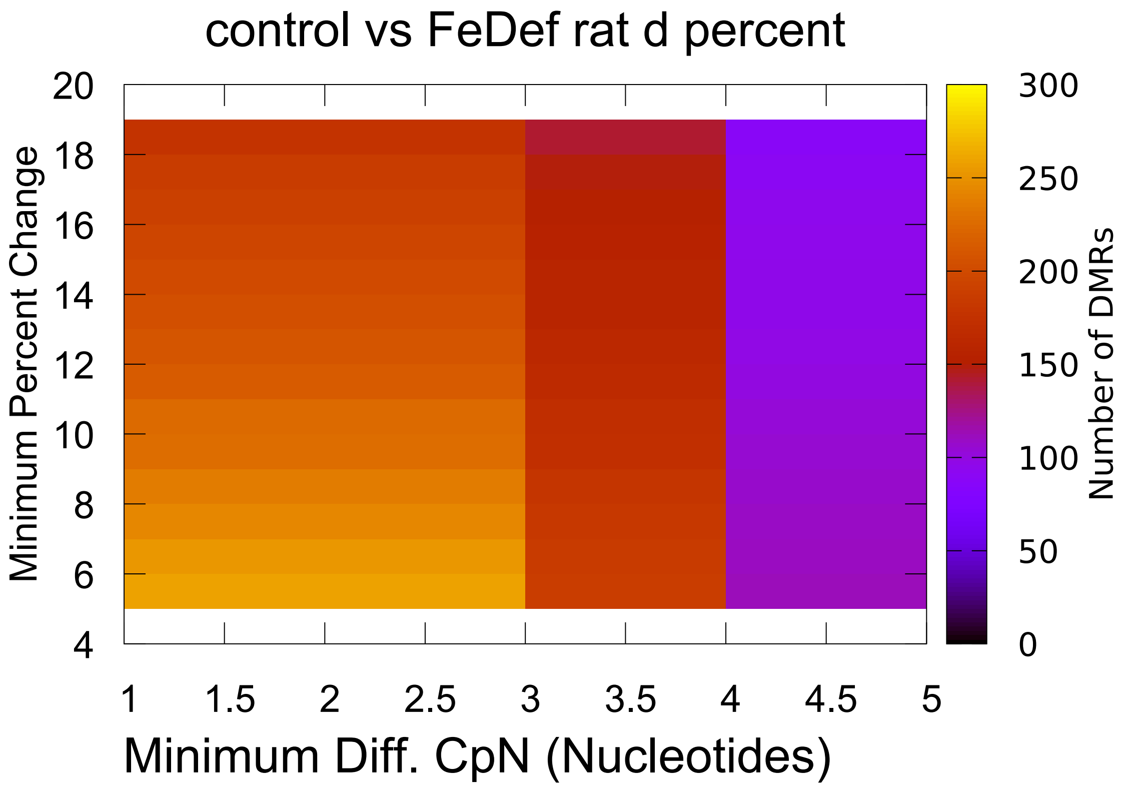

Figure S13: The minimum number of differentially methylated CpN “d”’ and the minimum percent change influence the number of DMRs found in the rat hippocampus. All other parameters held constant as indicated in Table S6. This plot is made with GNUPlot using the Perl script plot_results.pl which is included with Defiant.

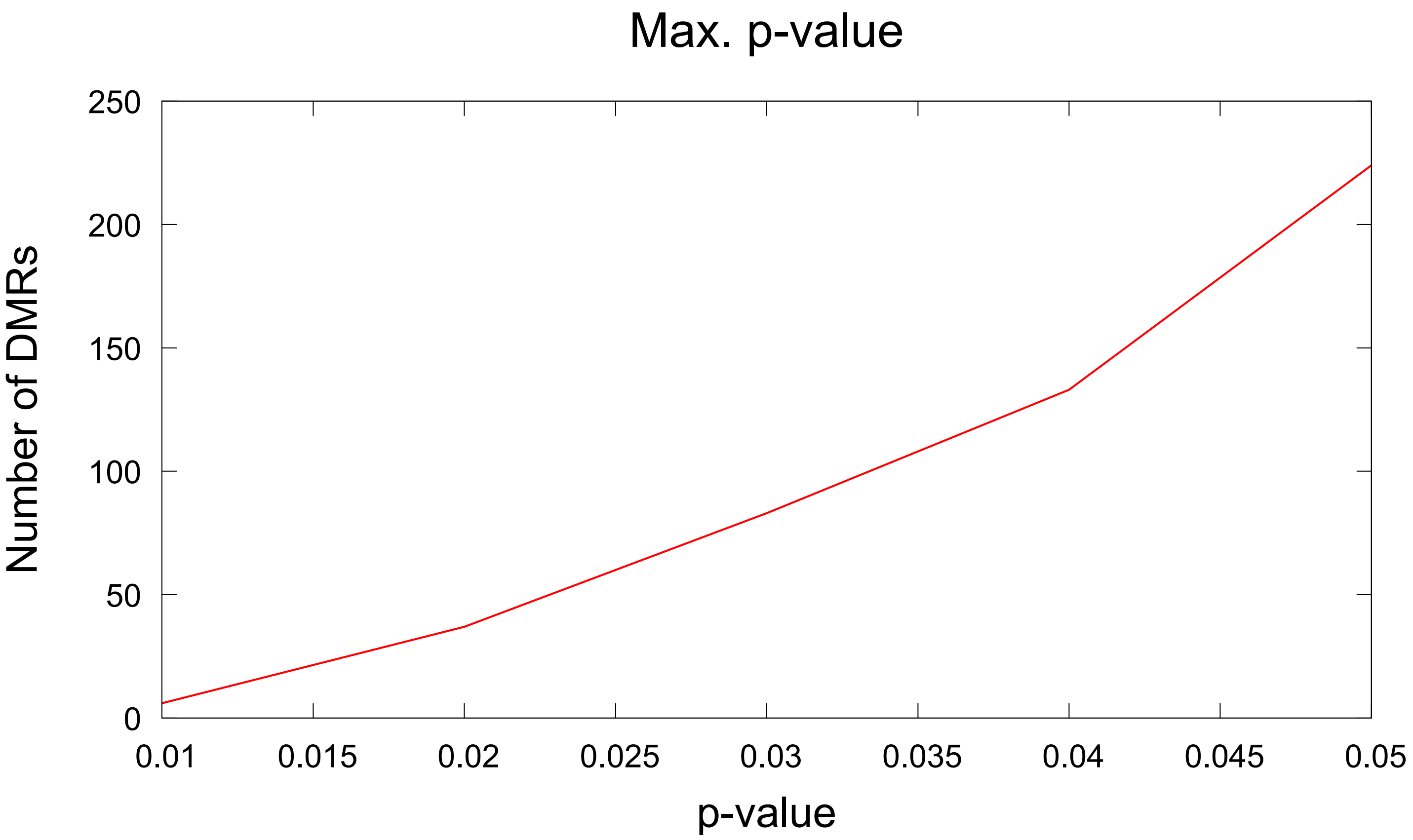

Figure S14: Maximum p-values display the expected behavior, with higher selectivity at lower p and higher sensitivity at higher p. All other parameters are held to values shown in Table S6.

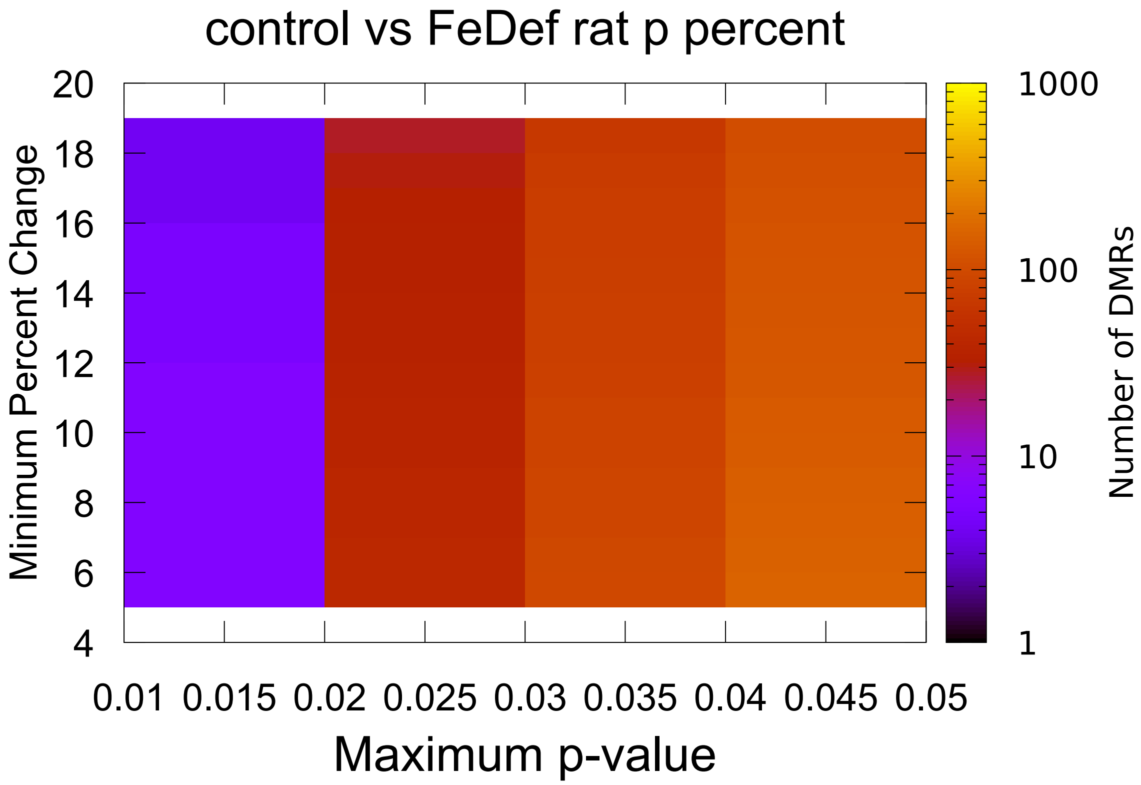

Figure S15: The maximum p-value and the minimum percent change influence the number of DMRs found in the rat hippocampus. The p-value appears to have a stronger influence on the number of DMRs found than the minimum percent cutoff. All other parameters held constant as indicated in Table S6. This plot is made with GNUPlot using the Perl script plot_results.pl which is included with Defiant.

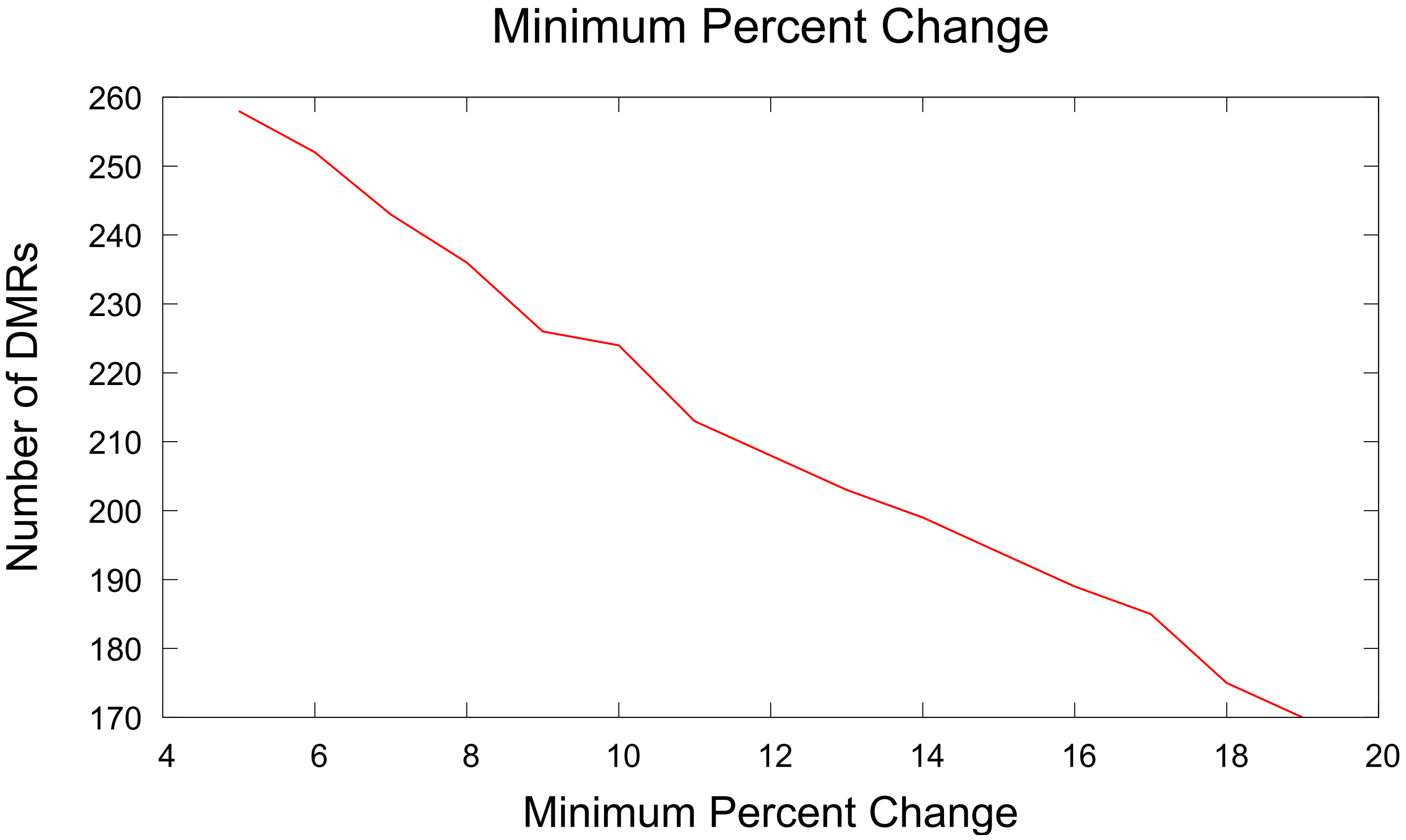

Figure S16: Minimum percent change influences the number of DMRs found. Larger minimum percent cutoffs show fewer DMRs, while there will be more DMRs showing smaller minimum percent cutoffs. All other parameters are held to values shown in Table S6.

References

[1] Beltran-Valero de Bernabé, D.; Voit, T.; Longman, C.; Steinbrecher, A.; Straub, V.; Yuva, Y.; Herrmann, R.; Sperner, J.; Korenke, C.; Diesen, C.; Dobyns, W. B.; Brunner, H. G.; van Bokhoven, H.; Brockington, M.; Muntoni, F. *J. Med. Genet.* **2004**, *41*(5), e61.

[2] Kong, R.; Shao, S.; Wang, J.; Zhang, X.; Guo, S.; Zou, L.; Zhong, R.; Lou, J.; Zhou, J.; Zhang, J.; Song, R. *Am. J. Med. Genet. Part B: Neuropsychiatric Genetics* **2016**, *171*(2), 203–208.

[3] Yoo, A. S.; Staahl, B. T.; Chen, L.; Crabtree, G. R. *Nature* **2009**, *460*, 642–646.

[4] Hota, S. K.; Bruneau, B. G. *Development* **2016**, *143*(16), 2882–2897.

[5] Nicolas, G.; Bennoun, M.; Devaux, I.; Beaumont, C.; Grandchamp, B.; Kahn, A.; Vaulont, S. *Proc. Natl. Acad. Sci. U.S.A.* **2001**, *98*(15), 8780–8785.

[6] Cocks, G.; Wilde, J. I.; Graham, S. J.; Bousgouni, V.; Virley, D.; Lovestone, S.; Richardson, J. *J. Alzheimers Dis.* **2010**, *19*(2), 631–646.

[7] Mastaitis, J.; Min, S.; Elvert, R.; Kannt, A.; Xin, Y.; Ochoa, F.; Gale, N. W.; Valenzuela, D. M.; Murphy, A. J.; Yancopoulos, G. D.; Gromada, J. *Proc. Natl. Acad. Sci. U.S.A.* **2015**, *112*(6), 1845–1849.

[8] Schiavone, S.; Jaquet, V.; Sorce, S.; Dubois-Dauphin, M.; Hultqvist, M.; Backdahl, L.; Holmdahl, R.; Colaianna, M.; Cuomo, V.; Trabace, L.; Krause, K.-H. *Transl Psychiatry* **2012**, *2*.

[9] Guerrieri, D.; Praag, H. *Oncotarget* **2015**, *6*(21).

[10] Liu, Q.; Puche, A. C.; Wang, J. B. *Neurochem. Res.* **2008**, *33*(7), 1263–1276.

[11] Feldker, D. E. M.; Datson, N. A.; Veenema, A. H.; Meulmeester, E.; De Kloet, E. R.; Vreugdenhil, E. *Eur. J. Neurosci.* **2003**, *17*(2), 379–387.

[12] Achim, K.; Peltopuro, P.; Lahti, L.; Tsai, H.-H.; Zachariah, A.; Åstrand, M.; Salminen, M.; Rowitch, D.; Partanen, J. *BiO* **2013**.

[13] Thiesen, S.; Kübart, S.; Ropers, H.-H.; Nothwang, H. *Biochem. Biophys. Res. Commun.* **2000**, *273*(1), 364 – 369.

[14] Patil, S. P.; Tran, N.; Geekiyanage, H.; Liu, L.; Chan, C. *Neurosci. Lett.* **2013**, *554*, 121 – 125.

[15] Möller, A. *The protein SS18L1 is a potent suppressor of polyQ-mediated huntingtin aggregation and toxicity* PhD thesis, Universität zu Berlin, **2012**.

[16] Melcher, T.; Maas, S.; Herb, A.; Sprengel, R.; Higuchi, M.; Seeburg, P. H. *J. Biol. Chem.* **1996**, *271*(50), 31795–31798.

[17] Heinrich, J.; Proepper, C.; Schmidt, T.; Linta, L.; Liebau, S.; Boeckers, T. *Neuroscience* **2012**, *221*, 86 – 95.

[18] Kitiratschky, V. B.; Behnen, P.; Kellner, U.; Heckenlively, J. R.; Zrenner, E.; Jägle, H.; Kohl, S.; Wissinger, B.; Koch, K.-W. *Hum. Mutat.* **2009**, *30*(8), E782–E796.

[19] Rosales-Hernandez, A.; Beck, K. E.; Zhao, X.; Braun, A. P.; Braun, J. E. A. *Cell Stress Chaperones* **2009**, *14*(1), 71–82.

[20] Baecker, T.; Mangus, K.; Pfaender, S.; Chhabra, R.; Boeckers, T. M.; Grabrucker, A. M. *BioMetals* **2014**, *27*(4), 715–730.

[21] Bobowski, M.; Harduin-Lepers, A.; Delannoy, P. In *Handbook of Glycosyltransferases and Related Genes*; Taniguchi, N., Honke, K., Fukuda, M., Narimatsu, H., Yamaguchi, Y., Angata, T., Eds.; Springer Japan: Tokyo, 2014; pages 767–780.

[22] Costa, L. G.; de Laat, R.; Dao, K.; Pellacani, C.; Cole, T. B.; Furlong, C. E. *Neurotoxicology* **2014**, *43*, 3 – 9.

[23] Zhang, Y.; Yu, X.; Ichikawa, M.; Lyons, J. J.; Datta, S.; Lamborn, I. T.; Jing, H.; Kim, E. S.; Biancalana, M.; Wolfe, L. A.; DiMaggio, T.; Matthews, H. F.; Kranick, S. M.; Stone, K. D.; Holland, S. M.; Reich, D. S.; Hughes, J. D.; Mehmet, H.; McElwee, J.; Freeman, A. F.; Freeze, H. H.; Su, H. C.; Milner, J. D. *J. Allergy Clin. Immunol.* **2014**, *133*(5), 1400 – 1409.e5.

[24] Wolstenholme, J. T.; Rissman, E. F.; Bekiranov, S. *Genes, Brain and Behavior* **2013**, *12*(2), 166–180.

[25] Su, Y. A.; Zhang, Q.; Su, D. M.; Tang, M. X. *Int. J. Biol. Sci.* **2011**, *7*(3), 308–322.

[26] Hagino, S.; Iseki, K.; Mori, T.; Zhang, Y.; Hikake, T.; Yokoya, S.; Takeuchi, M.; Hasimoto, H.; Kikuchi, S.; Wanaka, A. *Glia* **2003**, *42*(2), 130–138.

[27] Labonne, J. D. J.; Lee, K.-H.; Iwase, S.; Kong, I.-K.; Diamond, M. P.; Layman, L. C.; Kim, C.-H.; Kim, H.-G. *Hum. Genet.* **2016**, *135*(7), 757–771.

[28] Horvath, E. *Genetic Investigations And Counselling On Neurogenetic Disorders* PhD thesis, University of Szege, **2014**.

[29] Xia, Z.; Chibnik, L. B.; Glanz, B. I.; Liguori, M.; Shulman, J. M.; Tran, D.; Khoury, S. J.; Chitnis, T.; Holyoak, T.; Weiner, H. L.; Guttmann, C. R. G.; De Jager, P. L. *PLOS ONE* **2010**, *5*(11), 1–6.

[30] Cai, W. W.; Wang, L.; Chen, Y. *Biosci. Biotechnol. Biochem* **2010**, *74*(10), 2050–2055.

[31] Montague, P.; Barrie, J. A.; Thomson, C. E.; Kirkham, D.; McCallion, A. S.; Davies, R. W.; Kennedy, P. G. E.; Griffths¿, I. R. *European Journal of Neuroscience* **1998**, *10*(4), 1321–1328.

[32] Wurdak, H.; Zhu, S.; Romero, A.; Lorger, M.; Watson, J.; yuan Chiang, C.; Zhang, J.; Natu, V. S.; Lairson, L. L.; Walker, J. R.; Trussell, C. M.; Harsh, G. R.; Vogel, H.; Felding-Habermann, B.; Orth, A. P.; Miraglia, L. J.; Rines, D. R.; Skirboll, S. L.; Schultz, P. G. *Cell Stem Cell* **2010**, *6*(1), 37 – 47.

[33] Caracausi, M.; Rigon, V.; Piovesan, A.; Strippoli, P.; Vitale, L.; Pelleri, M. C. *Hippocampus* **2016**, *26*(1), 13–26.

[34] Lang, H.; Minaian, K.; Freudenberg, N.; Hoffmann, R.; Brandsch, R. *Biochem. J.* **1994**, *299*(2), 393–398.

[35] Matsumoto, K.; Ishii, N.; Yoshida, S.; Shiosaka, S.; Wanaka, A.; Tohyama, M. *Brain Res. Mol. Brain Res.* **1998**, *61*(1-2), 179 – 189.

[36] Properzi, F.; Carulli, D.; Asher, R. A.; Muir, E.; Camargo, L. M.; Van Kuppevelt, T. H.; Ten Dam, G. B.; Furukawa, Y.; Mikami, T.; Sugahara, K.; Toida, T.; Geller, H. M.; Fawcett, J. W. *Eur. J. Neurosci.* **2005**, *21*(2), 378–390.

[37] Salogiannis, J. *Regulation of excitatory synapse development by the RhoGEF Ephexin5* PhD thesis, Harvard University, **2013**.

[38] Iacobas, S.; Iacobas, D.; Spray, D.; Scemes, E. *Brain Res.* **2012**, *1487*, 131 – 139.

[39] Lu, L.; Wei, L.; Peirce, J. L.; Wang, X.; Zhou, J.; Homayouni, R.; Williams, R. W.; Airey, D. C. *BMC Genomics* **2008**, *9*(1), 444.

[40] Anta, B.; Martín-Rodríguez, C.; Gomis-Perez, C.; Calvo, L.; López-Benito, S.; Calderón-García, A. A.; Vicente-García, C.; Villarroel, Á.; Arévalo, J. C. *J. Biol. Chem.* **2016**, *291*(36), 19132–19145.

[41] Gillingham, A. K.; Sinka, R.; Torres, I. L.; Lilley, K. S.; Munro, S. *Developmental Cell* **2014**, *31*(3), 358 – 373.

[42] Gelbard, H. A.; Dewhurst, S.; Maggirwar, S. B.; Kiebala, M.; Polesskaya, O.; Gendelman, H. E. *Neurotherapeutics* **2010**, *7*(4), 392 – 398.

[43] Borgesius, N. Z.; van Woerden, G. M.; Buitendijk, G. H. S.; Keijzer, N.; Jaarsma, D.; Hoogenraad, C. C.; Elgersma, Y. *J. Neurosci.* **2011**, *31*(28), 10141–10148.

[44] Chen, L.-T.; Liang, W.-X.; Chen, S.; Chen, Z.; Chen, S.-J. *Blood* **2015**, *120*(21), 764–764.

[45] Kim, G. W.; Lin, J. E.; Waldman, S. A. *Trends in Endocrinology & Metabolism* **2013**, *24*(4), 165 – 173.

[46] Chong, A.; Zhang, Z.; Choi, K. P.; Choudhary, V.; Djamgoz, M. B.; Zhang, G.; Bajic, V. B. *Genomics* **2007**, *89*(3), 378 – 384.

[47] Jahanshad, N.; Rajagopalan, P.; Hua, X.; Hibar, D. P.; Nir, T. M.; Toga, A. W.; Jack, C. R.; Saykin, A. J.; Green, R. C.; Weiner, M. W.; Medland, S. E.; Montgomery, G. W.; Hansell, N. K.; McMahon, K. L.; de Zubicaray, G. I.; Martin, N. G.; Wright, M. J.; Thompson, P. M.; the Alzheimer's Disease Neuroimaging Initiative. *Proc. Natl. Acad. Sci. USA* **2013**, *110*(12), 4768–4773.

[48] Wilburn, B.; Rudnicki, D. D.; Zhao, J.; Weitz, T. M.; Cheng, Y.; Gu, X.; Greiner, E.; Park, C. S.; Wang, N.; Sopher, B. L.; Spada, A. R. L.; Osmand, A.; Margolis, R. L.; Sun, Y. E.; Yang, X. W. *Neuron* **2011**, *70*(3), 427 – 440.

[49] Szablewski, L. *J. Alzheimers Dis.* **2016**, *55*(4), 1307–1320.

[50] Shcherbatyy, V.; Carson, J.; Yaylaoglu, M.; Jäckle, K.; Grabbe, F.; Brockmeyer, M.; Yavuz, H.; Eichele, G. *Neuroinformatics* **2015**, *13*(1), 111–125.

[51] Suls, A.; Jaehn, J. A.; Kecskés, A.; Weber, Y.; Weckhuysen, S.; Craiu, D. C.; Siekierska, A.; Djémié, T.; Afrikanova, T.; Gormley, P.; von Spiczak, S.; Kluger, G.; Iliescu, C. M.; Talvik, T.; Talvik, I.; Meral, C.; Caglayan, H. S.; Giraldez, B. G.; Serratosa, J.; Lemke, J. R.; Hoffman-Zacharska, D.; Szczepanik, E.; Barisic, N.; Komarek, V.; Hjalgrim, H.; Møller, R. S.; Linnankivi, T.; Dimova, P.; Striano, P.; Zara, F.; Marini, C.; Guerrini, R.; Depienne, C.; Baulac, S.; Kuhlenbäumer, G.; Crawford, A. D.; Lehesjoki, A.-E.; de Witte, P. A.; Palotie, A.; Lerche, H.; Esguerra, C. V.; Jonghe, P. D.; Helbig, I.; Hendrickx, R.; Holmgren, P.; Stephani, U.; Muhle, H.; Pendiziwiat, M.; Appenzeller, S.; Selmer, K.; Brilstra, E.; Koeleman, B.; Rosenow, F.; Leguern, E.; Sterbova, K.; Magdalena, B.; Rodica, G.; Arsene, O. T.; Diana, B.; Guerrero-Lopez, R.; Ortega, L.; Todorova, A. P.; Kirov, A. V.; Robbiano, A.; Arslan, M.; Yiş, U.; Ivanović, V. *Am. J. Hum. Genet.* **2013**, *93*(5), 967 – 975.

[52] Kuppili, R. R.; Bose, K. In *Proteases in Apoptosis: Pathways, Protocols and Translational Advances*; Bose, K., Ed.; Springer International Publishing: Cham, 2015; pages 53–94.

[53] Marín, F.; Nieto, M. A. *Dev. Dynam.* **2006**, *235*(9), 2586–2591.

[54] Anthoni, H.; Zucchelli, M.; Matsson, H.; Müller-Myhsok, B.; Fransson, I.; Schumacher, J.; Massinen, S.; Onkamo, P.; Warnke, A.; Griesemann, H.; Hoffmann, P.; Nopola-Hemmi, J.; Lyytinen, H.; Schulte-Körne, G.; Kere, J.; Nøthen, M. M.; Peyrard-Janvid, M. *Hum. Mol. Gen.* **2007**, *16*(6), 667.

[55] Tweedie, D.; Rachmany, L.; Rubovitch, V.; Lehrmann, E.; Zhang, Y.; Becker, K. G.; Perez, E.; Miller, J.; Hoffer, B. J.; Greig, N. H.; Pick, C. G. *Experimental Neurology* **2013**, *239*, 170 – 182.

[56] Sperlágh, B.; Vizi, E. S. *Neurochemical Research* **2007**, *32*(11), 1978–1989.

[57] Dutta, S.; Dawid, I. B. *Development* **2010**, *137*(18), 3013–3018.

[58] Yoon, K.-J.; Lee, H.-R.; Jo, Y. S.; An, K.; Jung, S.-Y.; Jeong, M.-W.; Kwon, S.-K.; Kim, N.-S.; Jeong, H.-W.; Ahn, S.-H.; Kim, K.-T.; Lee, K.; Kim, E.; Kim, J.-H.; Choi, J.-S.; Kaang, B.-K.; Kong, Y.-Y. *Molecular Brain* **2012**, *5*(1), 40.

[59] Long, P.; Samnakay, P.; Jenner, P.; Rose, S. *Eur. J. Neurosci.* **2012**, *36*(6), 2733–2742.

[60] Bollen, E.; Prickaerts, J. *IUBMB Life* **2012**, *64*(12), 965–970.

[61] Gong, J.; Grodsky, J. D.; Zhang, Z.; Wang, P. *Eukaryotic Cell* **2014**, *13*(10), 1290–1299.

[62] Hernandez-Deviez, D. J.; Casanova, J. E.; Wilson, J. M. *Nat. Neurosci.* **2002**, *5.*

[63] Brown, M. E.; Bridgman, P. C. *J. Neurobiol.* **2004**, *58*(1), 118–130.

[64] Ishisaka, M.; Hara, H. *J Pharmacol Sci* **2014**, *124*(3), 336–343.

[65] Wong, T. H.; Chiu, W. Z.; Breedveld, G. J.; Li, K. W.; Verkerk, A. J. M. H.; Hondius, D.; Hukema, R. K.; Seelaar, H.; Frick, P.; Severijnen, L.-A.; Lammers, G.-J.; Lebbink, J. H. G.; van Duinen, S. G.; Kamphorst, W.; Rozemuller, A. J.; Bakker, E. B.; Neumann, M.; Willemsen, R.; Bonifati, V.; Smit, A. B.; van Swieten, J. *Brain* **2014**, *137*(5), 1361.

[66] DeAndrade, M. P.; Zhang, L.; Doroodchi, A.; Yokoi, F.; Cheetham, C. C.; Chen, H.-X.; Roper, S. N.; Sweatt, J. D.; Li, Y. *PLOS ONE* **2012**, *7*(4), 1–10.

[67] Fernández-Fernández, D.; Rosenbrock, H.; Kroker, K. S. *Synapse* **2015**, *69*(10), 484–496.

[68] Morderer, D.; Nikolaienko, O.; Skrypkina, I.; Cherkas, V.; Tsyba, L.; Belan, P.; Rynditch, A. *Gene* **2012**, *505*(2), 360 – 364.
